# Fecal Feline Coronavirus RNA Shedding and Spike Gene Mutations in Cats with Feline Infectious Peritonitis Treated with GS-441524

**DOI:** 10.3390/v14051069

**Published:** 2022-05-17

**Authors:** Marina L. Meli, Andrea M. Spiri, Katharina Zwicklbauer, Daniela Krentz, Sandra Felten, Michèle Bergmann, Roswitha Dorsch, Kaspar Matiasek, Martin Alberer, Laura Kolberg, Ulrich von Both, Katrin Hartmann, Regina Hofmann-Lehmann

**Affiliations:** 1 Clinical Laboratory, Department of Clinical Diagnostics and Services, Center for Clinical Studies, Vetsuisse Faculty, University of Zurich, CH-8057 Zurich, Switzerland; aspiri@vetclinics.uzh.ch (A.M.S.); rhofmann@vetclinics.uzh.ch (R.H.-L.); 2 Clinic of Small Animal Medicine, Centre for Clinical Veterinary Medicine, LMU Munich, D-80539 Munich, Germany; k.zwicklbauer@medizinische-kleintierklinik.de (K.Z.); d.krentz@medizinische-kleintierklinik.de (D.K.); s.felten@medizinische-kleintierklinik.de (S.F.); n.bergmann@medizinische-kleintierklinik.de (M.B.); r.dorsch@medizinische-kleintierklinik.de (R.D.); hartmann@lmu.de (K.H.); 3 Section of Clinical & Comparative Neuropathology, Institute of Veterinary Pathology, Centre for Clinical Veterinary Medicine, LMU Munich, D-80539 Munich, Germany; kaspar.matiasek@neuropathologie.de; 4 Division of Paediatric Infectious Diseases, Dr. von Hauner Children’s Hospital, University Hospital, LMU-Munich, D-80337 Munich, Germany; martin.alberer@lrz.uni-muenchen.de (M.A.); laura.kolberg@med.uni-muenchen.de (L.K.); ulrich.von.both@med.lmu.de (U.v.B.); 5 German Center for Infection Research (DZIF), Partner Site Munich, D-80337 Munich, Germany

**Keywords:** FIP, FCoV, shedding, viral loads, spike gene mutations, therapy, treatment, Xraphconn^®^, feces, sequencing, reinfection

## Abstract

As previously demonstrated by our research group, the oral multicomponent drug Xraphconn^®^ containing GS-441524 was effective at curing otherwise fatal feline infectious peritonitis (FIP) in 18 feline coronavirus (FCoV)-infected cats. The aims of the current study were to investigate, using samples from the same animals as in the previous study, (1) the effect of treatment on fecal viral RNA shedding; (2) the presence of spike gene mutations in different body compartments of these cats; and (3) viral RNA shedding, presence of spike gene mutations, and anti-FCoV antibody titers in samples of 12 companion cats cohabitating with the treated cats. Eleven of the eighteen treated FIP cats (61%) were shedding FCoV RNA in feces within the first three days after treatment initiation, but all of them tested negative by day 6. In one of these cats, fecal shedding reoccurred on day 83. Two cats initially negative in feces were transiently positive 1–4 weeks into the study. The remaining five cats never shed FCoV. Viral RNA loads in feces decreased with time comparable with those in blood and effusion. Specific spike gene mutations linked to systemic FCoV spread were consistently found in blood and effusion from treated FIP cats, but not in feces from treated or companion cats. A new mutation that led to a not yet described amino acid change was identified, indicating that further mutations may be involved in the development of FIP. Eight of the twelve companion cats shed FCoV in feces. All but one of the twelve companion cats had anti-FCoV antibodies. Oral treatment with GS-441524 effectively decreased viral RNA loads in feces, blood, and effusion in cats with FIP. Nonetheless, re-shedding can most likely occur if cats are re-exposed to FCoV by their companion cats.

## 1. Introduction

Feline coronaviruses (FCoVs) are enveloped RNA viruses with a single-stranded, almost 30-kb long, non-segmented genome of positive polarity. They belong to the family Coronaviridae; to the order Nidovirales; together with canine coronavirus (CCoV) and transmissible gastroenteritis virus (TEGV) of pigs, to the subfamily Coronavirinae; to the genus Alphacoronavirus; to the subgenus Tegacovirus and to the species Alphacoronavirus 1 [1]. FCoVs show high mutation rates upon replication, leading to the formation of viral quasispecies, and thus, multiple genetic virus variants related by mutations [2].

FCoVs are endemic in cats and the prevalence of infection is very high, up to 90%, especially in multi-cat environments [3,4,5,6]. FCoVs are transmitted horizontally via the fecal–oral route, leading to infection of enterocytes [7,8]. In most of the cases, enteric infection only induces mild enteritis, mostly without clinical signs. However, 4–5% of adult FCoV-infected cats and 5–10% of kittens in multi-cat environments develop feline infectious peritonitis (FIP) [9,10], a fatal FCoV-induced immune-mediated disease characterized by granulomatous vasculitis and perivasculitis [11]. FIP develops when highly virulent FCoVs (FIP-associated FCoVs) arise by mutations from less virulent FCoVs within an individual FCoV-infected cat [12]. This process starts with mutations conferring the ability to infect a broadened target cell spectrum, including monocytes/macrophages, allowing for systemic infection [13]. The key features of FIP development are the activation of infected monocytes/macrophages and the ability of the mutated strains to effectively and sustainably replicate in these target cells [14,15,16,17]. Systemically infected healthy cats can carry low amounts of virus in different organs [18,19]; however, different studies have shown that in cats suffering from FIP, viral replication in blood and viral loads in tissues are generally significantly higher [18,20,21,22].

Cats start shedding FCoV in feces as early as two days after experimental infection, and continuously shed for at least two weeks, after which shedding declines and becomes intermittent [8,19,23]. Upon natural infection, cats can either develop a persistent infection and shed the virus in feces continuously or intermittently for a long time, or eliminate the infection and stop virus shedding [8,24,25]. Furthermore, cats can become repeatedly infected with the same or a different virus strain, resulting in intermittent life-long shedding [19,26,27,28,29]. In multi-cat environments, the number of persistent shedders and the overall shedding frequency represent risk factors for the development of FIP in individual cats [7]. The enterocytes in the colon are the main site of FCoV persistence [23,25,29]. The virus can also be found in tissue macrophages, even in the absence of viremia. Therefore, in infected animals, viremia and FIP can develop even after clearance of the virus from the intestine [23].

Anti-FCoV antibodies are detected in the blood as early as one week after experimental infection [19,25,30]. However, antibodies do not seem to be able to confer immunity, as antibody-positive cats can be re-infected and/or develop FIP. High antibody titers are associated with FCoV shedding in feces [8,26,31,32]. Moreover, there is evidence of a positive correlation of shedding frequency as well as shedding intensity with antibody titers [4,33,34]. However, persistent FCoV shedders can also be antibody-negative, and on the other hand, antibody-positive cats can be negative in four sequentially collected fecal samples [4]. Furthermore, although in one study, all 15 included cats with FIP had high anti-FCoV antibody titers, there was much overlap with titers of cats that were presented for any reason to a veterinary hospital, so individual titers alone were of no value for the diagnosis of FIP [35].

Both viral genetic determinants and the host immune system are likely to play roles in the development of FIP [14,36,37], but once FIP is diagnosed, the outcome so far has always been fatal. Several antiviral and immunomodulating FIP treatment regimens have been investigated (for a comprehensive review, see [38]). Thus far, the most promising results were obtained using not yet licensed compounds: a 3C-like protease inhibitor and the nucleoside analog GS-441524 [39,40,41,42]. An unlicensed but commercially available oral multi-component drug called Mutian^®^, Mutian X^®^, or Xraphconn^®^ (Nantong Biotechnology, Nantong, China) containing the nucleoside analog GS-441524 [43] proved to be effective in stopping fecal FCoV shedding in naturally infected animals and was successfully applied together with feline interferon omega for the treatment of a cat with non-effusive FIP uveitis [44,45]. We recently published a prospective study using this orally formulated compound (Xraphconn^®^, Mutian Life Sciences Limited, Nantong, China) containing GS-441524 for the treatment of FIP. All 18 cats with FIP were cured and showed massive improvement in clinical and laboratory parameters within the first few days of treatment without serious adverse effects [43].

The aims of the present study were to further investigate the outcome of these 18 cats with FIP treated with Xraphconn^®^ [43] by (1) determining viral RNA shedding in feces during treatment; (2) comparing fecal viral RNA loads with those in other compartments, i.e., blood and effusion; and (3) comparing viral loads with the presence of anti-FCoV antibodies. Furthermore, viral RNA-positive samples were analyzed to detect FCoV spike gene mutations that lead to the amino acid substitution M1058L and S1060A [46]. Finally, samples from cats living together with the treated FIP cats (companion cats) were tested for viral RNA shedding (including determination of viral sequences) in feces and anti-FCoV antibodies in blood to further investigate the potential for re-exposure of treated cats.

## 2. Materials and Methods

### 2.1. In Vivo Study Design and Patients

Eighteen cats with FIP were enrolled in the prospective treatment study: inclusion criteria and study set-up were previously described in detail [43]. Cats were treated orally with Xraphconn^®^ (a multicomponent drug containing GS-441524 [43]) for 84 days (day 0 to day 83). The drug was applied at 5 mg/kg per os (PO) q24h in cats without neurological and/or ocular signs (low dose treatment; 16 cats) and at 10 mg/kg PO q24h in cats with neurological and/or ocular signs (high dose treatment; two cats). All cats were hospitalized for the first 8 days (day 0 to day 7). After discharge, the owners continued daily administration of the compound [43]. Cats were not allowed outdoors during the treatment period. However, some cats from the same households with close contact with the treated FIP cats (17/18 treated cats lived with between 1 and 9 companion cats) had outside access (Table 1). From some of these companion cats, a fecal sample and a serum sample were collected during or after the treatment period of the FIP cats to monitor for infection of these companion cats and virus shedding in the household (Table 1).

### 2.2. Sample Collection for Determination of Viral Loads and Anti-FCoV Antibody Titers

From the 18 treated FIP cats, blood and, if present and accessible, thoracic and abdominal effusions were collected on days 0, 2, 4, 7, 14, 28, 56, and 83 for viral RNA load determination. Serum samples were collected on days 0, 7, 14, 28, 56, and 83 to measure anti-FCoV antibodies, as described previously [43]. In addition, for the present study, fecal samples were collected from the 18 treated FIP cats on days 0, 1, 2, 3, 4, 5, 6, 7, 14, 28, 56, and 83. Both voided fecal samples and fecal swabs were collected. Fecal swabs were taken if no voided feces were available.

In companion cats, fecal samples for viral RNA loads and serum samples for antibody titer quantification were collected once at different time points (Table 1). All samples were stored at −80 °C until analysis.

### 2.3. Anti-FCoV Antibody Titers

Serum samples from treated FIP cats and companion cats were analyzed by an indirect immunofluorescence assay (IFA) as previously described [47,48,49]. Cat samples were tested at dilutions of 1:25, 1:100, 1:400, 1:1600, and 1:6400. A positive control (aliquoted serum sample from an anti-FCoV antibody-positive field cat) and a negative control (aliquoted serum from a specific pathogen-free anti-FCoV antibody-negative cat) were run with each slide.

### 2.4. FCoV RNA Loads in Feces, Blood, and Effusions

FCoV RNA load was determined in blood, fecal, and effusion samples by RT-qPCR. Briefly, viral total nucleic acids (TNA) were extracted from 200 μL of effusion or 100 μL EDTA anti-coagulated whole blood or 200 µL of fecal samples. For fecal samples, depending on the collected material, approximately 0.05 g of feces was dissolved in 1 mL of 1X sterile phosphate buffered saline, pH 7.4 (PBS; Gibco, Life Technologies Ltd., Paisley, UK), or fecal swabs were dissolved in 400 μL of PBS; fecal material was processed as described [19]. TNA were extracted using the MagNA Pure 96 (Roche Diagnostics AG, Rotkreuz, Switzerland) and the MagNA Pure 96 DNA and Viral NA SV Kit (Roche Diagnostics) according to the manufacturer’s instructions, with an elution volume of 100 µL. For all samples, the viral NA plasma external lysis SV 4.0 protocol was applied. For each batch of extractions, negative controls were run in parallel to check for cross-contamination.

A previously published real-time RT-qPCR assay was used to detect the FCoV 7b gene [50]. The methods were adapted as described previously [43]. All RT-qPCR were run with 5 μL of TNA in a final volume of 25 μL. Positive and negative PCR controls were run in parallel using an ABI7500Fast instrument (Applied Biosystems, Thermo Fisher Scientific, Waltham, MA, USA). Fecal samples were run neat and diluted 1:5 in nuclease free water to detect possible RT-qPCR inhibition. An FCoV RNA standard curve was run in parallel to determine the viral RNA copy number. The FCoV RNA standard was produced as follows: a NotI linearized pCR™II-TOPO^®^ TA vector containing the sequence of the target assay was in vitro transcribed using the Large Scale SP6 Transcription Kit (Novagen, Juro supply, Lucerne, Switzerland), followed by purification by the RNeasy Mini Kit (Qiagen AG, Hombrechtikon, Switzerland). In vitro transcribed RNA was quantified, the RNA copy number was calculated as described [51], and 10-fold serial dilutions were prepared in PCR-grade water with 30 µg/mL of carrier rRNA (Sigma-Aldrich, Merk, Darmstadt, Germany). The RNA was stored in 50 µL aliquots at −80 °C until use.

### 2.5. Sanger Sequencing to Detect Spike Gene Mutations in Fecal Samples, Blood, and Effusions

FCoV RT-qPCR-positive samples underwent conventional RT-PCR amplifying part of the spike gene potentially containing FCoV mutations that lead to protein substitutions M1058L and S1060A [46]. Shortly, the FCoV-UCD1-S.3022 forward and FCoV-UCD1-S.3636 reverse primer were used for a first amplification (amplicon 615 bp) using the one-step RT-PCR Kit (SuperScript III RT/Platinum Taq Mix, Invitrogen, Thermo Fisher Scientific). The reaction composition and cycling conditions were used as published previously [52]. For the second nested PCR step, if needed, a modified primer pair amplifying the region of interest (amplicon length 134 bp), FCoV-UCD1-S.3027f and FCoV-UCD1-S.3160r, and the Phusion Hot Start II High-Fidelity DNA Polymerase (Thermo Fisher Scientific) were used as described [52]. Gel electrophoresis was performed with 1.5% agarose gels containing 0.1 mM GelRed (Biotium, Hayward, ON, Canada). Following the addition of Orange G loading dye (Bioconcept, Allschwil, Switzerland) in a 1:5 ratio to the amplified DNA, samples were loaded on the gels and run at 100 V. A 1-kilobase-pair DNA ladder (Fermentas, St. Leon-Rot, Germany), or alternatively, a Gene Ruler DNA Ladder Mix (Thermo Fisher Scientific), was used for molecular size comparisons. Appropriate bands were cut out with sterile razor blades and weighed. PCR amplicons were purified with the MinElute or QIAquick Gel extraction Kit (Qiagen) according to the manufacturer’s instructions and sequenced directly. Selected nested PCR amplicons were cloned into the pCR^®^II-TOPO plasmid with the TOPO TA Cloning Kit for Sequencing (Invitrogen, Thermo Fisher Scientific) using TOP 10 competent cells according to the manufacturer’s instructions, after adding A-overhangs using the Taq DNA Polymerase (Sigma-Aldrich). The reaction mix for A-tailing contained 10 μL of PCR product, 1.2 μL Taq Buffer (10×), 0.24 μL dATP (10 mM), 0.1 μL Taq DNA Polymerase (5 U/μL), and 0.46 μL RNase-free water and was incubated at 72 °C for 15 min. Transformed TOP 10 cells were grown overnight at 37 °C on Luria broth plates containing ampicillin. From each cloned RT-PCR amplicon, 10 colonies were picked and cultured overnight at 37 °C in Luria-Bertani liquid medium containing ampicillin. Cultures were centrifuged at 6800× *g* for 3 min and the pellets were used for further manipulation. Plasmids were isolated from the TOP 10 cell pellets with the QIAprep Miniprep Kit (Qiagen) according to the manufacturer’s instructions. Concentrations of the eluted DNA were determined using a Nanodrop 2000c (NanoDrop products, Wilmington, NC, USA). Sanger sequencing was performed using M13 forward and M13 reverse primers on 1.2 μg plasmid DNA. Sequencing was performed in a commercial lab (Microsynth, Balgach, Switzerland). Nucleotide sequences were manually edited, assembled, aligned and compared to sequences from Genbank using Geneious Prime^®^ (https://www.geneious.com (accessed on 16 May 2022); Biomatters Ltd., Auckland, New Zealand). Evolutionary analyses were conducted using the Molecular Evolutionary Genetics Analysis software package version X (MEGA X) [53]. Nucleotide sequences were aligned using the ClustalW algorithm [54]. Bootstrap phylogenetic trees were constructed using the Minimum Evolution method [55] and Jukes–Cantor model. A bootstrap analysis was performed to test the stability of the trees with 1000 replicates [56]. Sequences are shown as a FASTA file in the Supplementary Material.

### 2.6. Statistics

Frequencies of treated FIP cats presenting with effusions, shedding viral RNA in feces, and being RT-qPCR-positive in blood at different time points of the study, or of treated FIP cats and companion cats having high anti-FCoV antibody titers (≥1 > 1:6400) were compared using Fisher’s exact test (p_Fisher_). FCoV RNA loads in fecal swabs and voided fecal samples were compared using the non-parametric Mann–Whitney U–test. Viral RNA copy number differences in animals between selected treatment days were compared using the non-parametric Wilcoxon signed-rank test (p_W_). The correlation of viral loads in the three compartments (feces, blood, and effusions) and antibody titers was calculated using the nonparametric Spearman rank correlation (p_S_; r = correlation coefficient). A *p*-value < 0.05 was considered to be statistically significant in all cases. Statistical analysis was performed using the GraphPad Prism 9.3.1 software (GraphPad software, LLC, San Diego, CA, USA).

## 3. Results

### 3.1. Treatment Study

**Figure 1:**
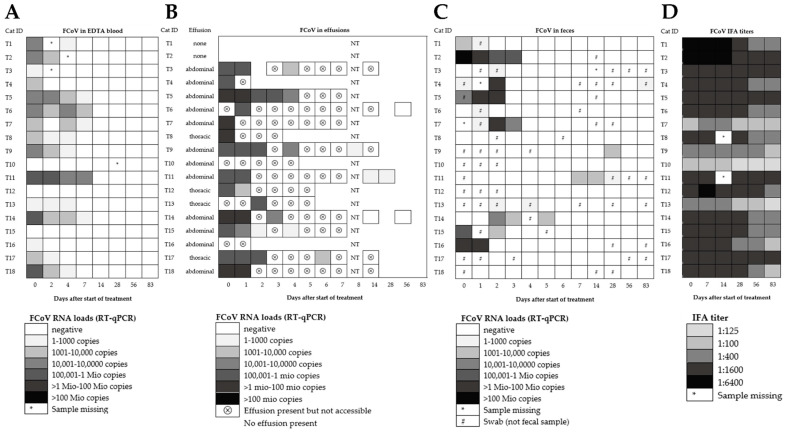
Feline coronavirus (FCoV) viral RNA loads in different compartments and anti-FCoV titers of treated FIP cats (adapted from [43]). (**A**) EDTA anti-coagulated blood; (**B**) Effusions; (**C**) Voided fecal samples or fecal swabs; and (**D**) Antibody titers. FCoV RNA loads were determined by quantitative reverse transcription polymerase chain reaction (RT-qPCR) (**A**–**C**). Antibody titers were determined by indirect immunofluorescence assay (IFA). NT, not tested.

Clinical outcomes of successful treatment in cats T1–18 have been described in detail previously [43]. Briefly, all cats survived, demonstrating a full clinical recovery within 84 days after treatment initiation [43]. In all cats, clinical (Karnofsky score, body temperature, and body weight) and laboratory parameters (hematocrit, lymphocyte count, bilirubin, total protein, albumin, globulin, and serum amyloid A concentrations) improved constantly and significantly within the first few days of treatment [43]. The number of cats with effusion had decreased significantly by day 14 after treatment initiation, in comparison to day 0 (p_Fisher_ = 0.0045; Figure 1B, Table 2). On day 83, effusion was no longer present in any of the cats.

### 3.2. Fecal FCoV RNA Shedding and Viral Loads in Treated FIP Cats

**Figure 2:**
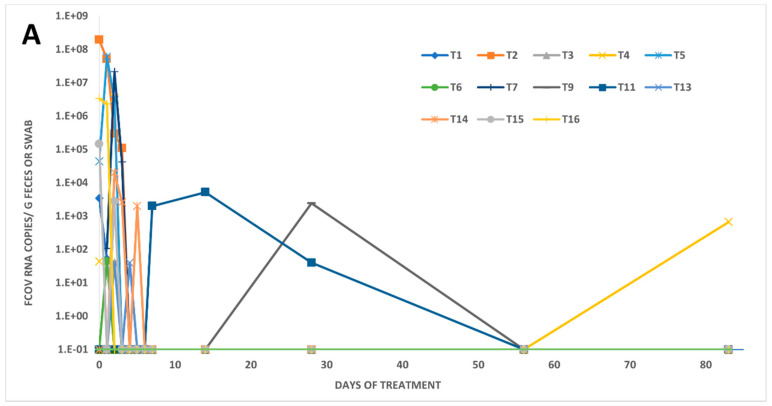

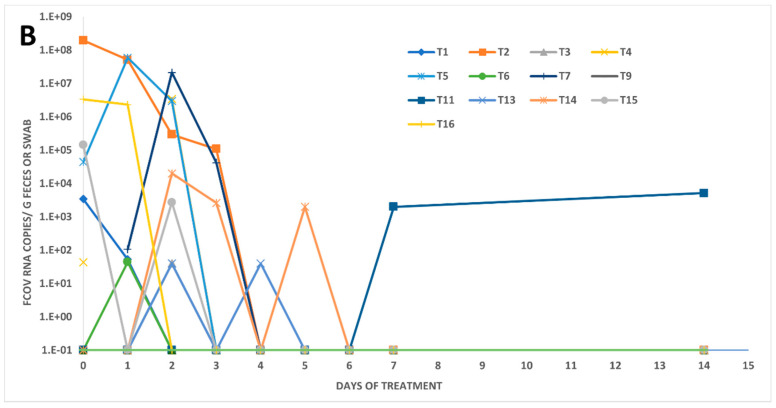
Viral RNA loads in voided fecal samples and fecal swabs of FIP treated cats (T1–T18). Fecal samples were collected from the 18 treated FIP cats (**A**): on days 0, 1, 2, 3, 4, 5, 6, 7, 14, 28, 56, and 83; (**B**): on days 0, 1, 2, 3, 4, 5, 6, 7 and 14. Both voided fecal samples and fecal swabs were collected. Fecal swabs were taken if no voided feces were available. RNA loads were measured by feline coronavirus (FCoV) quantitative reverse transcription polymerase chain reaction (RT-qPCR). Values are given in copy numbers per g feces or swab.

In five of the eighteen treated FIP cats (28%), fecal shedding could neither be detected prior to treatment (day 0) nor at any timepoint throughout the entire study (cats T8, T10, T12, T17, T18; Figure 1C). Only 6/18 cats (33%) tested RT-qPCR-positive in feces on day 0. However, when samples from the first three days of the study were included in the analysis (day 0 to day 2), about 2/3 of the treated FIP cats (11/18; 61%) were positive for FCoV RNA in feces. Fecal virus shedding had stopped in all of these 11 initially shedding cats by day 6, but one cat tested positive later again on day 83 (cat T4). Moreover, two cats (cats T11 and T9) that initially had not shed any viral RNA became RT-qPCR-positive on days 7, 14, and 28 (T11), and on day 28 (T9), respectively. The number of cats shedding FCoV RNA in feces had significantly decreased by day 4 when compared to day 0 (p_Fisher_ = 0.0408). Interestingly, in seven cats, viral loads initially increased during treatment (T3, T4, T5, T6, T7, T13, T14), while in three cats (T1, T2, T16), the loads decreased. Moreover, in one cat (T15), the RNA load first decreased and then rebounded before RNA shedding ceased (Figure 2).

Viral RNA loads in voided fecal samples ranged from almost 196 million copies/g feces on day 0 in cat T2 to 2000 copies/g feces in cat T11 (on day 7) and cat T14 (on day 5). In fecal swabs, loads were lower than in voided fecal samples; loads in swabs were as high as 44,000 copies per swab on day 0 (cat T5) to as low as 40 copies per swab on days 2 (cats T3 and T13) and 4 (cat T13). The influence of the different sample collection methods (swabs versus voided fecal samples) was assessed by comparing the resulting FCoV RNA loads per PCR reaction; thereby, the RNA loads in fecal swabs were significantly lower than those in voided fecal samples (p_MWU_ = 0.0069).

### 3.3. FCoV RNA Loads in Other Body Compartments of Treated FIP Cats

**Figure 3:**
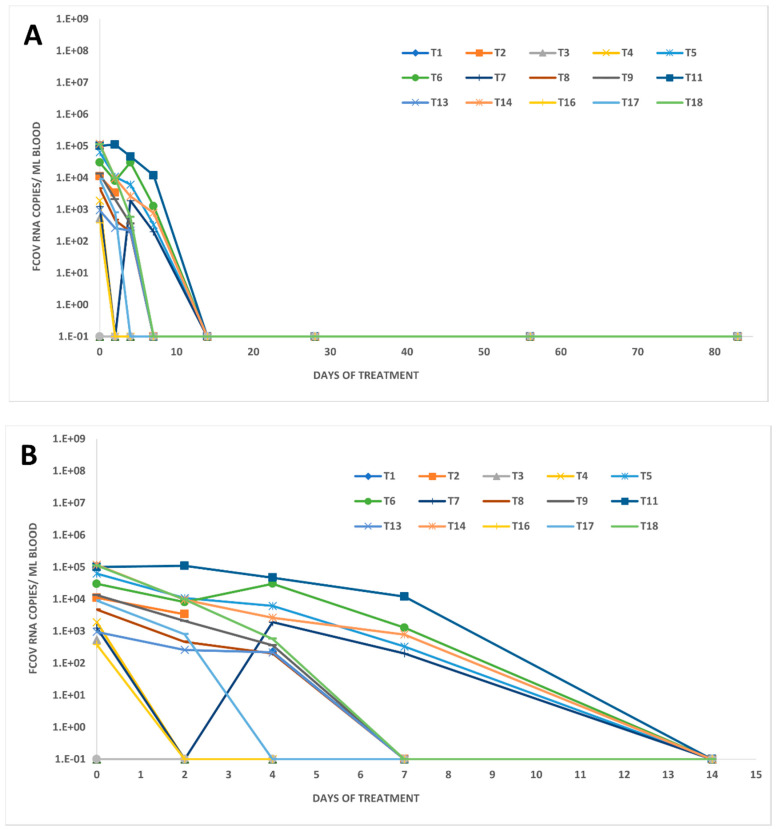
Viral RNA loads in blood samples of treated FIP cats. EDTA blood was collected (**A**): on days 0, 2, 4, 7, 14, 28, 56, and 83 and (**B**): on days 0, 2, 4, 7, and 14 for the determination of viral RNA loads. Loads were measured by feline coronavirus (FCoV) quantitative reverse transcription polymerase chain reaction (RT-qPCR) in cats. Values are given in copy numbers per mL blood.

Remarkably, in most of the 18 treated FIP cats (15/18; 83%), viral RNA was detected in blood before the start of the treatment (day 0), but by day 7, the number of cats positive for viral RNA in blood had significantly decreased (p_Fisher_ = 0.0020). Blood viral RNA loads in 14 of these 15 cats decreased even earlier than viral loads in feces, by day 2 after treatment initiation (Figure 1A and Figure 3). Blood viral RNA loads ranged from almost 120,000 to about 400 copies per mL blood on day 0. Loads significantly decreased from day 0 to day 2 (p_W_ = 0.0061; values in 2/16 cats missing and thus two cats excluded; *n* = 16 cats) and from day 0 to day 7 (p_W_ < 0.0001; *n* = 18 cats). In two of the positive cats with an initial decrease (cats T6 and T7), the blood viral RNA load rebounded transiently on day 4 to the same level as on day 0, but decreased again thereafter (Figure 3). The last viral RNA-positive samples were found on day 7. By day 14, no viral RNA was detected in any of the cats (Figure 1A), indicating that all cats had cleared viral RNAemia by that time.

**Figure 4:**
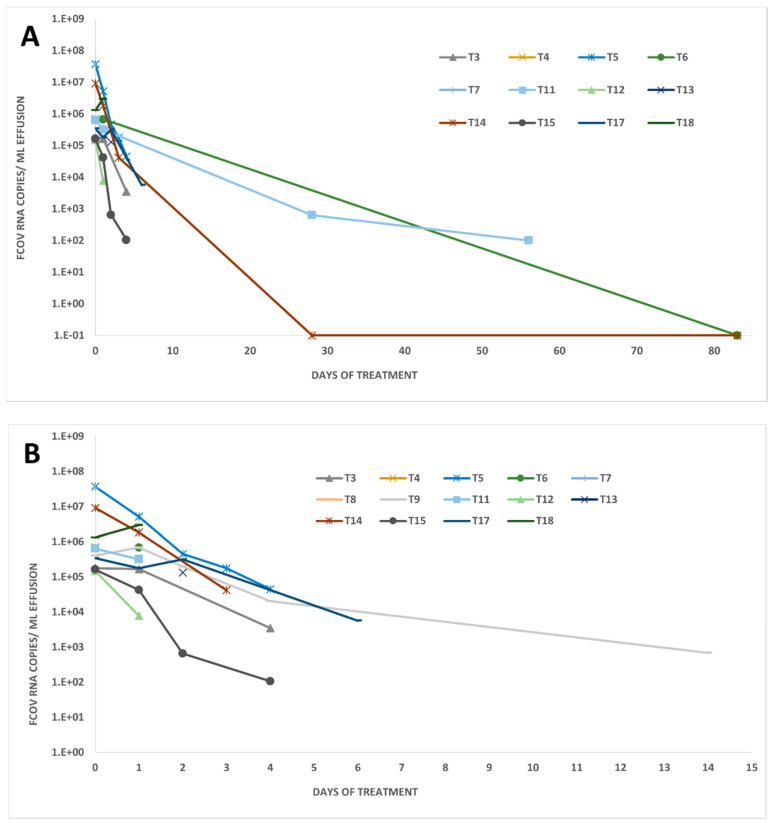
Viral RNA loads in accessible effusions of treated FIP cats. Present and accessible thoracic and abdominal effusions were collected (**A**): on days 0, 2, 4, 7, 14, 28, 56, and 83 and (**B**): on days 0, 2, 4, 7, and 14 for viral RNA load determination. Loads were measured by feline coronavirus (FCoV) quantitative reverse transcription polymerase chain reaction (RT-qPCR) in cats with accessible effusions (*n* = 16). Values are given in copy numbers per mL effusion. When no value is depicted, either effusions were not (or no longer) accessible or no effusion was present.

Sixteen of the eighteen treated FIP cats had effusion at treatment initiation (day 0), and all accessible effusions (*n* = 12) tested RT-qPCR-positive at that time (Figure 1B). Viral RNA load in effusion decreased rapidly in most cats, while only two cats (T9 and T18) had increased loads one day after treatment initiation (Figure 1B and Figure 4). After the initial increase, viral RNA loads in the effusion of cat T9 decreased from day 1 (684,114 copies/mL) to the last effusion sampling on day 8 (691 copies/mL). The effusion of cat T18 was only accessible on day 0 and day 1. Therefore, it remains unknown if the viral RNA load would have decreased over time in this cat.

Generally, viral RNA was detectable in all but three accessible effusion samples (from cat T6 on day 56 and cat T14 on days 14 and 56), and two of the three had a volume smaller than the 200 µL usually used for TNA extraction. To increase the sensitivity of detection, these three samples were retested by RT-qPCR with 10 replicates, yielding negative results.

There was a good correlation between viral RNA loads in blood and effusion (p_S_ = 1.3 × 10^−8^; r = 0.856; 95% CI 0.6990–0.9340). No correlation was found between viral RNA loads in feces and effusion or blood, respectively.

### 3.4. Anti-FCoV Antibody Titers in Treated FIP Cats

All treated FIP cats were antibody-positive at the beginning of the study (day 0), most of them with high antibody titers (14/18 with titers ≥ 1:1600). Anti-FCoV antibody titers declined in 14/18 cats, in some cats starting as early as 28 days after treatment initiation (Figure 1D). In four cats (T3, T5, T11, T17), titers remained unchanged throughout the observation period. In only one cat (T8), a slight titer increase was observed after one week of treatment (Figure 1D). No correlation was found between fecal RNA loads and antibody titers (p_S_ = 0.06; r = 0.187; 95% CI 0.0120–0.3710). A very weak correlation between blood RNA loads and antibody titers was found (p_S_ = 0.037; r = 0.204; 95% CI 0.0071 to 0.3856).

### 3.5. Spike Gene Mutations in Fecal Samples, Blood, and Effusions in Treated FIP Cats

Only samples with a cycle threshold (Ct) below 35 (corresponding to a viral RNA load of approximately 1850 copies per mL in blood and effusion samples or 20,200 copies per g in fecal samples) were considered for sequencing (Table 2).

**Table 2:** Sequencing results of the viral spike gene including positions 1058 and 1060 in fecal samples, blood, and effusion of treated FIP cats.

	Effusions					Blood				Fecal Samples										
Cat Number	d0	d1	d2	d3	d4	d0	d2	d4	d7	d0	d1	d2	d3	d4	d5	d7	d14	d28	d56	d83
T1						nb		x	n	x	x	n	n	n	n	n	n	n	n	n
T2						nb	nb		n	**MS**	**MS**	nb	nb	n	n	n	n	n	n	n
T3	nb	**LS**			nb	x		n	n	n	n	x	n	n	n	n		n	n	n
T4	**LS**					x	n	n	n	x		**MS**	n	n	n	n	n	n	n	x
T5	**LS**	**LS**	**LS**	nb	nb	nb	nb	nb	x	x	**MS**	**MS**	n	n	n	n	n	n	n	n
T6		nb				nb	nb	nb	x	n	x	n	n	n	n	n	n	n	n	n
T7	**LS**					x	n	nb	x		x	**MS**	**MS**	n	n	n	n	n	n	n
T8	**LS**					nb	x	x	n	n	n	n	n	n	n	n	n	n	n	n
T9	nb	nb	nb		nb	nb	nb	x	n	n	n	n	n	n	n	n	n	x	n	n
T10						n	n	n	n	n	n	n	n	n	n	n	n	n	n	n
T11	**FS** *	**FS** *				nb	nb	nb	nb	n	n	n	n	n	n	x	x	x	n	n
T12	**MA** *	nb				n	n	n	n	n	n	n	n	n	n	n	n	n	n	n
T13			nb			x	x	x	n	n	n	x	n	x	n	n	n	n	n	n
T14	**MA** *	**MA** *		nb		**MA** *	nb	nb	x	n	n	nb	x	n	x	n	n	n	n	n
T15	**LS**	nb	x		x	n	n	n	n	**MS**	n	x	n	n	n	n	n	n	n	n
T16						x	n	n	n	**MS**	**MS**	n	n	n	n	n	n	n	n	n
T17	nb	nb	nb			nb	x	n	n	n	n	n	n	n	n	n	n	n	n	n
T18	**LS**	**LS**				nb	nb	x	n	n	n	n	n	n	n	n	n	n	n	n

Bold: sequenced samples. LS: leucine at position 1058 and serine at position 1060; MS: methionine at position 1058 and serine at position 1060; FS: phenylalanine at position 1058 and serine at position 1060; MA: leucine at position 1058 and alanine at position 1060; nb: sample with no band detected after nested RT-PCR; x: FCoV RT-qPCR-positive but not sequenced due to low viral RNA load; n: FCoV RT-qPCR-negative sample; gray shading: no sample available. * Confirmed by cloning of the PCR amplicon and sequencing of several clones.

Of the 30 RT-qPCR-positive fecal samples of treated FIP cats (from days 0 to 83), 18 samples qualified for sequencing (Ct < 35), of which 10 could be sequenced successfully. In all 10 sequenced fecal samples, collected between days 0 and 3 of the study from six treated FIP cats, the amino acid sequence corresponded to the FCoV wild-type, with a methionine at position 1058 and a serine at position 1060 of the S protein (MS).

Of the 40 RT-qPCR-positive blood samples of treated FIP cats (from days 0 to 7), 23 qualified for sequencing (Ct < 35), but only one sample from cat T14 collected on day 0 was sequenced successfully. The spike protein in this sample had a methionine at position 1058 but an alanine instead of a serine at position 1060 (MA).

Of the 33 RT-qPCR-positive effusion samples of treated FIP cats (from days 0 to 4), 31 qualified for sequencing (Ct < 35); of these, 15 samples, collected on days 0 to 2 from 10 treated FIP cats were sequenced successfully. In seven out of these ten cats (T3, T4, T5, T7, T8, T15, T18), the amino acid leucin instead of a methionine was found at position 1058 of the S protein (LS; Table 2). In two of the cats (T12 and T14), an alanine was found instead of a serine at position 1060 (MA), and in one cat (T11), an atypical mutation at position 1058 was detected, in that the methionine was replaced by the amino acid phenylalanine (FS; Table 2). In cat T14, in which the viral spike gene from both effusion and blood could be sequenced, the same mutation was found (MA) in both compartments.

Sequencing results were confirmed in cats T11 (effusion from days 0 and 1), T12 (effusion from day 0), and T14 (effusion from days 0 and 1, and blood from day 0) by cloning of the PCR product and subsequent sequencing. All sequences could be confirmed without exception using at least nine clones in the effusion samples of cats T11 (days 0 and 1), T12 (day 0), and T14 (day 0); three clones in the effusion sample of cat T14 (day 1); and six clones in the blood sample of cat T14 (day 0).

### 3.6. Fecal FCoV Shedding, Spike Gene Mutations, and Anti-FCoV Antibody Titers in Companion Cats

All companion cats were clinically healthy at the time of sample collection. Eight of twelve (67%) companion cats were positive for viral RNA in feces (Table 3). The percentage of cats shedding viral RNA in feces was not significantly different between companion cats (67%; samples collected at various time points) and treated FIP cats at the start of the treatment (61%; days 0 to 2; Figure 1C). FCoV viral RNA loads in companion cats ranged from almost 75 million copies/g feces (C16) on day 56 to 160,000 copies/g feces (C15 on day 56) in voided fecal samples and from 347,000 copies/fecal swab (C11B on day 83) to about 2000 copies/fecal swab (C11A on day 83).

All companion cats were antibody-positive (one with a borderline antibody titer; Table 3). The antibody titers of the companion cats (in samples collected at various time points) ranged from 1:25 to 1:1600. Significantly fewer companion cats had titers of ≥1:1600 (2/11) compared to the treated FIP cats on day 0 (17/18 cats; p_Fisher_ = 0.0054).

Four of eight positive fecal samples from companion cats were successfully sequenced. In all samples, the amino acid sequence corresponded to the wild-type, with a methionine at position 1058 and a serine at position 1060 of the S protein (MS; Table 3).

### 3.7. Phylogenetic Analysis of Sequences of Treated FIP Cats and Companion Cats

**Figure 5:**
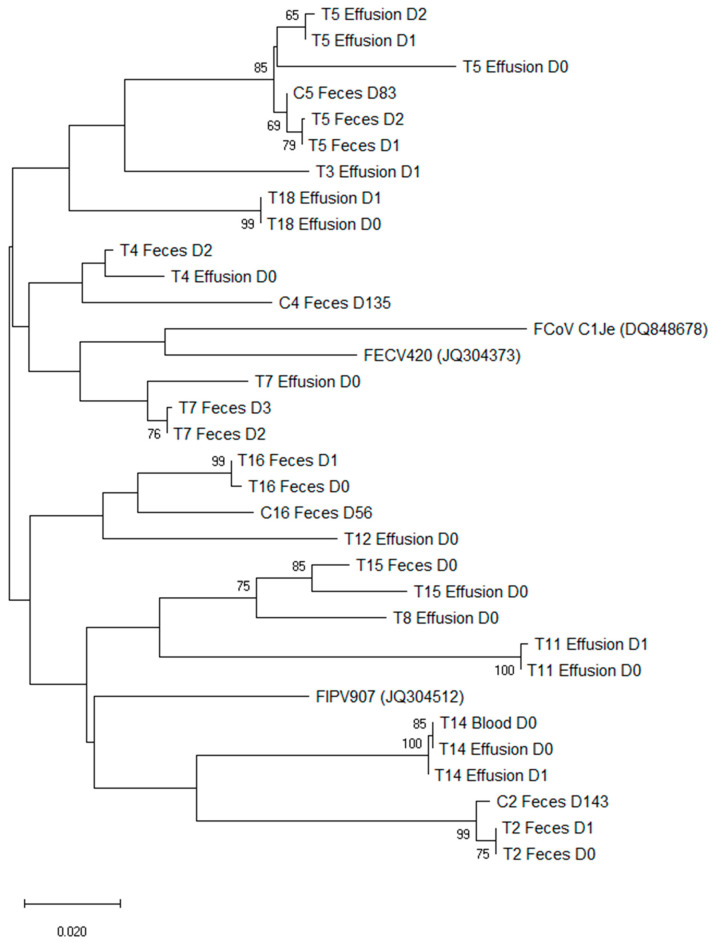
Bootstrap phylogenetic tree of spike (S) gene sequences. The evolutionary history was inferred using the Minimum Evolution (ME) method [55]. The optimal tree is shown. The percentages of replicate trees in which the associated taxa clustered together in the bootstrap test (1000 replicates) are shown next to the branches (values below 60 are not displayed) [56]. The tree is drawn to scale, with branch lengths in the same units as those of the evolutionary distances used to infer the phylogenetic tree. The evolutionary distances were computed using the Jukes–Cantor method and are in the units of the number of base substitutions per site. The ME tree was searched using the Close Neighbor Interchange (CNI) algorithm [57] at a search level of one. The Neighbor Joining algorithm [58] was used to generate the initial tree. This analysis involved 86 nucleotide sequences. All ambiguous positions were removed for each sequence pair (pairwise deletion option). In total, there were 155 positions in the final dataset. Evolutionary analyses were conducted in MEGA X [53]. Each sequenced FCoV strain is defined by the cat ID (T: treated FIP cat; C: companion cat), the compartment from which it has been isolated (feces, blood, effusion), and day of treatment (D#). Three prototype FCoV sequences from Genbank have been used in the tree: an enteric FCoV pathotype (FECV420, Accession no.: JQ304373) and an FIP virus pathotype (FIPV907, Accession no.: JQ304512) from the study [46], and an FIP virus pathotype isolated from the jejunum of an FIP cat (FCoV C1Je, Accession no.: DQ848678).

A phylogenetic analysis of the sequenced 143 bp of the spike gene showed that sequences isolated from each individual animal clustered together (Figure 5). Fecal samples of the companion cats clustered together with the corresponding treated FIP cats.

## 4. Discussion

This prospective controlled treatment trial in field cats with a confirmed or highly suspected diagnosis of FIP clearly demonstrated that oral treatment with the nucleoside analog GS-441524, contained in the multicomponent drug Xraphconn^®^, decreased viral RNA loads not only in blood and effusion (where accessible) but also in feces within a short period of time after treatment initiation, and no cats were viremic after day 14 of treatment [43]. These findings demonstrate a highly effective response to the treatment. In addition, antibody titers decreased during the therapy. The current study renders more granular insights into RNA loads and the presence of spike gene mutations in different compartments of treated FIP cats, adding new data on fecal RNA shedding in these cats, and, in addition, collected information on fecal RNA shedding and antibody titers of cats cohabiting with the treated FIP cats.

The majority of the treated FIP cats in the present study (72%) shed viral RNA at some time point throughout the study, particularly during the early days of treatment; only five cats never tested RT-qPCR-positive in fecal samples. Interestingly, on day 0, only 8/18 cats shed FCoV although all had FIP. This observation is not surprising, as in a previous study, no fecal shedding could be detected in the majority of cats with FIP [59]. Nonetheless, in another study, FIP cats were more likely to shed viral RNA in feces compared to cats without FIP [60]. Another previous study found some evidence that an intact FCoV ORF3c is required for intestinal replication [61].

Fecal viral RNA loads decreased significantly during early treatment, and most of the cats had stopped shedding FCoV RNA as early as day 4 of the treatment. Elimination of virus shedding through treatment with the same compound (containing GS-441524) has been described earlier in a study in breeding and rescue catteries with FCoV infection (that did not have FIP) that aimed to reduce the infection pressure in multi-cat households by stopping fecal FCoV shedding [45]. In this study, the authors could prove that a dose of 4 mg/kg q24 h for four days was optimal to stop fecal FCoV RNA shedding in 95% of naturally FCoV-infected, healthy persistent shedders from five households. These results could be reproduced in the present study, where animals were treated with a slightly higher dose (5 mg/kg, with the exception of T1 and T2 that suffered from neurological and/or ocular signs and therefore received a dose of 10 mg/kg) and stopped FCoV shedding by day 4 of treatment. In the study of Addie et al., 2020, in some animals, FCoV shedding restarted upon re-infection or relapse as soon as three days after treatment stopped. In one cat (T4) from the present study, reoccurrence of shedding was detected 83 days after initiation of the treatment and 80 days after shedding had initially ceased in this cat. Moreover, in two cats (T9 and T11), no shedding was detected initially but then was detected later on during the treatment period. Two of these three cats (T4 and T9) started shedding after they had been discharged from the veterinary hospital. These two cats had companion animals in the same household. In the case of cat T4, there was one companion cat (C4), which was sampled at a later time point (day 135), in which the fecal swab tested RT-qPCR-positive. Cat T9 had three companion cats, but no samples were available from those cats. Nonetheless, it is likely that cats T4 and T9 were both re-infected at home by their feline companions during the treatment period and the positive fecal samples in the treated FIP cats most probably represented a superinfection with an intestinal FCoV from their companion cats. The absence of viremia in the treated cats at the time point of positive testing of their fecal samples supports this assumption. Moreover, clustering of the S gene sequence of viruses from T4 and the companion cat C4 was observed; this further supports the likelihood of a re-infection of the treated FIP cat by its companion cat in the same household. The same could be valid for cat T9, which had two companion cats in the same household. The third cat that started re-shedding (T11) was still in the hospital at that time (day 7). Therefore, it seems reasonable to assume that in this cat, the virus most likely originated from a sanctuary compartment within the cat. A superinfection is rather unlikely (although not completely excluded), since the cat was kept separately from other animals in the hospital. Further insight could be gained by the sequencing of the virus shed by cat T11 and its companion cat; unfortunately, the fecal samples from cat C11 could not be sequenced due to a low viral load.

Intermittent fecal FCoV shedding has been frequently described [8,24]. The fact that a cat during treatment with the nucleoside analog GS-441524 and re-exposure to FCoV could start (re-)shedding the virus demonstrates that treatment does not prevent infection of enterocytes. The concentration of the antiviral in the enterocytes is probably not high enough to prevent re-infection. On the other hand, enterocytes have a very short lifespan [62], which could explain the intermittent shedding (disappearance of cells infected with low pathogenic FCoV once the mutated virus has reached the monocyte/macrophage compartments, and re-infection of new enterocytes). Thus, intestinal FCoV infections in cats should not be treated with GS-441524 or other antiviral compounds, as this poses the risk of mutations and development of resistant virus strains, since up to 90% of cats in multi-cat households are infected with and shed FCoV in their feces [3,4,5,6], and re-infections occur continuously and cannot be prevented. Even if a multi-cat environment is cleared from FCoV, it is likely that FCoV will be reintroduced into the household within a short time [45]. Furthermore, a resistant viral strain could persist in an animal. If this animal later develops FIP the antiviral drug would not be efficient anymore. Additionally, resistant FCoV strains could be shed and transmitted to other cats.

A remarkably high percentage of cats with FIP in this study (83%) were viral RNA-positive in blood before treatment with relatively high viral RNA loads. FCoV viremia can last from about 7–21 days following experimental infection and then decline so that in cats with FIP, often no viral RNA is found in the blood [23,37]. On the other hand, a study in shelter cats detected FCoV RNA in the blood of healthy and sick cats without FIP [63]. In many other studies, detection of viral RNA in blood was a rather rare event in cats with confirmed FIP [64,65,66,67], making the sensitivity of FCoV RT-qPCR from blood for diagnosis of FIP very low. In addition, the specificity of FCoV RT-qPCR from blood was questioned by the fact that healthy FCoV-infected animals were also sometimes viral RNA-positive in blood [19,63]. Thus, the notion that viremia will likely be over by the time clinical signs of FIP appear [23,37] might have to be reconsidered in view of the current data. However, systemically infected healthy animals without FIP that were RT-qPCR-positive in blood had lower FCoV viral RNA loads than cats with FIP [19,21,30]. Consequently, a positive RT-qPCR from blood alone cannot be recommended for the diagnosis of FIP, but positive results with a high FCoV load can be used to support a diagnosis, and based on the data of the present study, FCoV RT-qPCR from blood could be a valuable diagnostic tool, especially in cats in which no effusion is present.

In 12 out of the 18 treated FIP cats, effusion was present and accessible. In all 12 effusion samples collected on day 0, high viral RNA loads were detected. This result alone supports the utility of the FCoV RNA detection in effusion for the diagnosis of FIP. Previous studies could also amplify FCoV RNA from most (72–100%) of the effusion samples collected from cats with FIP and none from cats without FIP [64,66,68,69]. Few exceptions have been published in which effusion samples from cats with other underlying diseases had positive results for FCoV RNA. Therefore, high FCoV RNA loads in effusion samples, particularly those that also show cytological and biochemical features suggestive of FIP, are highly supportive of FIP. The viral RNA loads in accessible abdominal or thoracic effusions decreased in all cats over the study period. In most cats, the effusion disappeared or was not accessible anymore before negative RT-qPCR results could be achieved. However, looking at the blood and fecal viral RNA loads, it is assumed that viral RNA was also cleared from the thoracic or abdominal compartment. Therefore, GS-441524 treatment was beneficial in reducing viral RNA loads in effusions, blood, and feces and cured abdominal and/or thoracic effusions in FIP-diseased cats. A good correlation of FCoV RNA loads between blood and effusions was found in the present study, indicating, as expected, exchange between two compartments.

Most cats in the present study with FIP had remarkably high antibody titers, which decreased in most but not all cats over time. It has been known for a long time that specific antibodies are responsible for enhanced FCoV uptake and replication in macrophages by Fc receptors, contributing to clinical manifestations through an Arthus-type reaction [70,71,72]. Therefore, by reducing the viral loads and thus antibody titers, treatment could also contribute to reducing this immunological reaction and, consequently, the clinical signs in sick cats. A correlation of anti-FCoV antibody titers with the amount and frequency of fecal viral shedding has been previously described [4,33,34]. This was not the case in this study. There were animals with high antibody titers and high viral loads in feces (T2, T4, T5, T14, T15, T16) but more animals with high antibody titers and low to no RNA fecal loads (T1, T3, T6, T8, T11, T12, T17, T18). Conversely, a cat (T7) with relatively low antibody titers shed a high amount of FCoV RNA in feces. Three cats (T9, T10, and T13) had both low antibody titers and low amounts or no viral RNA shedding. These results should lead to reconsideration of the value of measuring the antibody titers for evaluating FCoV fecal shedding; although in many cases, high antibody titers correlate with the likelihood and frequency of FCoV shedding and fecal viral loads, and chronic shedders have higher antibody titers and shed more virus [4], but this is not always the case. Therefore, antibody measurement cannot replace fecal RT-qPCR.

Decreasing antibody titers might be a sign of decreasing viral loads in the cats under therapy, although in the current study only a weak correlation was found between viral RNA loads and antibody titers. Antibody titers are expected to decrease slower than viral loads, since the half life of antibodies can be several weeks, while viral loads are immediately influenced by an effective antiviral therapy. Another factor that might contribute to a slower or no decrease in antibody titers might be continuous antigen stimulation by reinfection with FCoV shedding companion cats in the same household. Of the five cats with high antibody titers on day 83, three cats (T2, T5, T11) had at least one companion cat in the household and all of them tested positive in voided feces or fecal swabs collected on either day 83 or day 143.

The spike protein mediates entry of FCoV into host cells through receptor binding and membrane fusion [73,74]. A full genomic sequencing approach identified a mutation in a nucleotide position that was associated with an amino acid change in the putative fusion peptide of the FCoV S protein [46]. The majority of FCoV isolates from cats with FIP were found to carry this mutation [46,75]. However, according to other studies, the amino acid changes in the spike protein were supposed to be a hallmark of monocyte/macrophage tropism acquisition allowing systemic spread of the virus [60,76]. Nonetheless, not all tissue-derived viral sequences carried this mutation in healthy infected cats [52]. Sequencing of the region of the spike gene encoding for the S protein region containing the mutations M1058L and S1060A [46] revealed a clear tropism of the different strains. In fecal samples of both treated and companion cats of the present study, only the wild-type form with the amino acids methionine (M) and serine (S) was found. On the contrary, in sequenced effusion and blood samples, only the mutated form was detected. These results, together with the results of another study from our group using the same molecular methods in which FCoV RNA detected in various tissues and body fluids of cats without FIP did not contain the M1058L and S1060A S protein mutations [77], support the former hypothesis [46] that these mutations are hallmarks of FIP. Therefore, sequencing of the spike gene would certainly help to diagnose FIP. In most of the systemic samples, the combination of the amino acids leucine (L) and serine (S) was present, whereas, in the samples of cats T12 and T14, the variant with the amino acids methionine (M) and alanine (A) was present. Interestingly, in one animal (T11), mutations in the genomic sequence led to a not yet described amino acid change with phenylalanine (M1058F). This indicates that the previously described mutations in the spike gene [46] are not the only mutations that can be involved in the development of FIP. Many RT-qPCR-positive samples could not be sequenced in the present study. In general, the lower the viral load, the more difficult it is to obtain a PCR band even when using the nested RT-PCR protocol. A further reason that could prevent amplification of the target sequence is possible sequence variation hampering the primer binding. It was already noticed in other studies that this sequence analysis method only detects mutations in type I FCoVs and not type II FCoVs [60,75]. It would have been interesting to assess viral evolution and antiviral effects on it with a next-generation sequencing approach.

Phylogenetic analysis of the S gene sequences showed a clear clustering of samples coming from the same household, irrespective of the compartment from which they were isolated (feces, blood, or effusions) and of the pathotype (low pathogenic FCoVs or FIP-causing virus). Although the interpretation of this phylogenetic analysis is limited by the short length of the amplicons (about 143 bp) and by the fact that not all samples could be sequenced due to the low viral loads, these results confirm once more the validity of internal mutation theory over the theory of pathogenic strains. The internal mutation theory assumes that FIP causing viruses arise de novo from mutations of low pathogenic FCoV in infected animals, as demonstrated by the phylogenetic clustering of FIP-causing viruses and low pathogenic FCoVs according to geographic distribution rather than clustering according to pathotype [12,52,78,79].

One limitation of the study is that in some cases, voided fecal samples were available, whereas in others, especially after hospital release, fecal swabs were collected. Viral RNA loads per PCR reaction in swabs of treated FIP cats were statistically significantly lower than in voided samples. The collection of voided samples was sometimes impossible since in multi-cat households, it is often difficult to assign collected voided samples to an individual cat with certainty. On the other hand, in cases of swab collection, the risk of an insufficient amount of sample material collected is high. This condition could also have contributed to the absence of correlation between viral RNA loads in feces with those in blood, effusions, and antibody titers, possibly due to an insufficient amount of sample material in swabs. Therefore, if possible, it would be best for future studies to collect both voided and swab samples, but if only one is possible, it should preferably be a voided fecal sample. One could think that a further study limitation was the lack of a control group with untreated FIP cats. However, no control group was chosen purposely. All cats with FIP lacking appropriate treatment either die or have to be euthanized within a few days of diagnosis; the median survival time of untreated cats is only eight to nine days [80,81]. Therefore, for animal welfare and ethical reasons, a control group of FIP cats without treatment was not included in the study.

## 5. Conclusions

This study in cats with FIP demonstrated that oral treatment with GS-441524 effectively decreased viral RNA loads in feces, blood, and effusion of treated animals. Nonetheless reinfection through FCoV-shedding companion cats is likely to occur.

Viral RNA loads in blood correlated with viral RNA loads in effusion but neither one correlated with RNA loads in feces. In addition, no correlation was found between fecal RNA loads and antibody titers in this study, probably due to the fast antiviral effect of GS-441524 on fecal viral shedding but not on antibody titers.

We identified in one animal a mutation in the genomic sequence that led to a not yet described amino acid change with phenylalanine (M1058F), indicating that further mutations may be involved in the development of FIP. Phylogenetic analysis of S gene sequences showed that FCoV strains cluster according to the household and not the pathotype.

## Figures and Tables

**Table 1:** Cats treated in the study (T1–T18), signalment, signs associated with feline infectious peritonitis (FIP), and information on companion cats in the household (C1–C18) (table partially adapted from [43]).

Identification of Treated FIP Cats	Age (Months)	Sex ^1^	FIP-Associated Cardinal Signs ^2^	Number of Companion Cats in the Same Household	Identification of Companion Cats with Samples Available	Collection Time Point (Day of Collection) ^3^ of Fecal and Serum Samples from Companion Cats
T1	6	mn	OS	1	C1 ^4^	145
T2	6	mi	NS + OS	1	C2	143
T3	10	mn	AE	1		
T4	7	mi	AE	1	C4	135
T5	6	fi	AE	1	C5	83
T6	11	mn	AE	1		
T7	5	mi	AE + TE	1		
T8	6	mi	TE	0		
T9	9	mn	AE	3		
T10	39	fn	AE	3		
T11	57	fn	AE	3	C11A, C11B, C11C	83
T12	12	mn	TE	1	C12	83
T13	29	fi	TE	9		
T14	8	mi	AE	1	C14	56
T15	8	mi	AE	1	C15	56
T16	9	fn	AE	1	C16	56
T17	8	mi	TE	1		
T18	8	fi	AE	2	C18	56

^1^ mn: male neutered; mi: male intact; fn: female neutered; fi: female intact. ^2^ OS: ocular signs; NS: neurological signs; AE: abdominal effusion; TE: thoracic effusion. ^3^ Days after treatment start in treated FIP cats. ^4^ Companion cats were labeled “C” and with the number of the corresponding treated cat. For cats C1, C2, and C4, samples were only available after the end of the treatment of cats T1, T2, and T4.

**Table 3:** Fecal viral RNA loads and anti-FCoV antibody titers in companion cats.

Companion Cat Number	Corresponding Treated FIP Cat	Day of Sample Collection in Companion Cats	Fecal Samples Collected	Fecal RNA Loads (Copies per g Feces or per Swab)	Sequencing Result	Anti-FCoV Antibody Titer
C1	T1	145	Swab	n	n	1:1600
C2	T2	143	Feces	66,337,219	**MS**	1:100
C4	T4 ^1^	135	Swab	27,545	**MS**	1:400
C5	T5	83	Feces	12,990,572	**MS**	1:1600
C11A	T11 ^2^	83	Swab	1906	nb	1:100
C11B	T11 ^2^	83	Swab	346,600	nb	1:400
C11C	T11 ^2^	83	Swab	178,345	nb	1:400
C12	T12	83	Feces	n	n	1:100
C14	T14	56	Swab	n	n	1:25 *
C15	T15	56	Feces	159,536	nb	1:400
C16	T16	56	Feces	74,714,117	**MS**	1:100
C18	T18	56	Swab	n	n	1:100

For better comparison, fecal swab samples are highlighted in gray. Bold: sequenced samples. ^1^ T4 showed reoccurrence of fecal shedding on day 83. ^2^ T11 was one of two cats that showed new appearance of fecal shedding during the study (day 7). FIP: Feline Infectious peritonitis; FCoV: Feline Coronavirus; g: gram; MS: methionine at position 1058 and serine at position 1060; nb: sample with no band detected after nested RT-PCR; n: FCoV RT-qPCR-negative sample. * Borderline titer.

## Data Availability

The authors confirm that all data analyzed in the study are available from the corresponding author upon reasonable request.

## Supplementary Materials

The following are available online at https://www.mdpi.com/article/10.3390/v14051069/s1, Table S1: Nucleotide sequences of the study in FASTA format.

## References

[1] Gonzalez J.M., Gomez-Puertas P., Cavanagh D., Gorbalenya A.E., Enjuanes L. (2003). A comparative sequence analysis to revise the current taxonomy of the family Coronaviridae. Arch. Virol..

[2] Lauring A.S., Andino R. (2010). Quasispecies theory and the behavior of RNA viruses. PLoS Pathog..

[3] Drechsler Y., Alcaraz A., Bossong F.J., Collisson E.W., Diniz P.P. (2011). Feline coronavirus in multicat environments. Vet. Clin. N. Am. Small Anim. Pract..

[4] Felten S., Klein-Richers U., Hofmann-Lehmann R., Bergmann M., Unterer S., Leutenegger C.M., Hartmann K. (2020). Correlation of Feline Coronavirus Shedding in Feces with Coronavirus Antibody Titer. Pathogens.

[5] Klein-Richers U., Hartmann K., Hofmann-Lehmann R., Unterer S., Bergmann M., Rieger A., Leutenegger C., Pantchev N., Balzer J., Felten S. (2020). Prevalence of Feline Coronavirus Shedding in German Catteries and Associated Risk Factors. Viruses.

[6] Pedersen N.C. (2009). A review of feline infectious peritonitis virus infection: 1963–2008. J. Feline Med. Surg..

[7] Foley J.E., Poland A., Carlson J., Pedersen N.C. (1997). Risk factors for feline infectious peritonitis among cats in multiple-cat environments with endemic feline enteric coronavirus. J. Am. Vet. Med. Assoc..

[8] Pedersen N.C., Allen C.E., Lyons L.A. (2008). Pathogenesis of feline enteric coronavirus infection. J. Feline Med. Surg..

[9] Addie D., Belak S., Boucraut-Baralon C., Egberink H., Frymus T., Gruffydd-Jones T., Hartmann K., Hosie M.J., Lloret A., Lutz H. (2009). Feline infectious peritonitis. ABCD guidelines on prevention and management. J. Feline Med. Surg..

[10] Addie D.D., Toth S., Murray G.D., Jarrett O. (1995). Risk of feline infectious peritonitis in cats naturally infected with feline coronavirus. Am. J. Vet. Res..

[11] Kipar A., May H., Menger S., Weber M., Leukert W., Reinacher M. (2005). Morphologic features and development of granulomatous vasculitis in feline infectious peritonitis. Vet. Pathol..

[12] Vennema H., Poland A., Foley J., Pedersen N.C. (1998). Feline infectious peritonitis viruses arise by mutation from endemic feline enteric coronaviruses. Virology.

[13] Pedersen N.C., Boyle J.F., Floyd K., Fudge A., Barker J. (1981). An enteric coronavirus infection of cats and its relationship to feline infectious peritonitis. Am. J. Vet. Res..

[14] Dewerchin H.L., Cornelissen E., Nauwynck H.J. (2005). Replication of feline coronaviruses in peripheral blood monocytes. Arch. Virol..

[15] Kipar A., Meli M.L. (2014). Feline infectious peritonitis: Still an enigma?. Vet. Pathol..

[16] Malbon A.J., Michalopoulou E., Meli M.L., Barker E.N., Tasker S., Baptiste K., Kipar A. (2020). Colony Stimulating Factors in Early Feline Infectious Peritonitis Virus Infection of Monocytes and in End Stage Feline Infectious Peritonitis; A Combined In Vivo And In Vitro Approach. Pathogens.

[17] Stoddart C.A., Scott F.W. (1989). Intrinsic resistance of feline peritoneal macrophages to coronavirus infection correlates with in vivo virulence. J. Virol..

[18] Gunn-Moore D.A., Gruffydd-Jones T.J., Harbour D.A. (1998). Detection of feline coronaviruses by culture and reverse transcriptase-polymerase chain reaction of blood samples from healthy cats and cats with clinical feline infectious peritonitis. Vet. Microbiol..

[19] Meli M., Kipar A., Muller C., Jenal K., Gonczi E., Borel N., Gunn-Moore D., Chalmers S., Lin F., Reinacher M. (2004). High viral loads despite absence of clinical and pathological findings in cats experimentally infected with feline coronavirus (FCoV) type I and in naturally FCoV-infected cats. J. Feline Med. Surg..

[20] Hornyak A., Balint A., Farsang A., Balka G., Hakhverdyan M., Rasmussen T.B., Blomberg J., Belak S. (2012). Detection of subgenomic mRNA of feline coronavirus by real-time polymerase chain reaction based on primer-probe energy transfer (P-sg-QPCR). J. Virol. Methods.

[21] Kipar A., Baptiste K., Barth A., Reinacher M. (2006). Natural FCoV infection: Cats with FIP exhibit significantly higher viral loads than healthy infected cats. J. Feline Med. Surg..

[22] Simons F.A., Vennema H., Rofina J.E., Pol J.M., Horzinek M.C., Rottier P.J., Egberink H.F. (2005). A mRNA PCR for the diagnosis of feline infectious peritonitis. J. Virol. Methods.

[23] Kipar A., Meli M.L., Baptiste K.E., Bowker L.J., Lutz H. (2010). Sites of feline coronavirus persistence in healthy cats. J. Gen. Virol..

[24] Addie D.D., Schaap I.A., Nicolson L., Jarrett O. (2003). Persistence and transmission of natural type I feline coronavirus infection. J. Gen. Virol..

[25] Vogel L., Van der Lubben M., te Lintelo E.G., Bekker C.P., Geerts T., Schuijff L.S., Grinwis G.C., Egberink H.F., Rottier P.J. (2010). Pathogenic characteristics of persistent feline enteric coronavirus infection in cats. Vet. Res..

[26] Addie D.D., Jarrett O. (2001). Use of a reverse-transcriptase polymerase chain reaction for monitoring the shedding of feline coronavirus by healthy cats. Vet. Rec..

[27] Foley J.E., Poland A., Carlson J., Pedersen N.C. (1997). Patterns of feline coronavirus infection and fecal shedding from cats in multiple-cat environments. J. Am. Vet. Med. Assoc..

[28] Harpold L.M., Legendre A.M., Kennedy M.A., Plummer P.J., Millsaps K., Rohrbach B. (1999). Fecal shedding of feline coronavirus in adult cats and kittens in an Abyssinian cattery. J. Am. Vet. Med. Assoc..

[29] Herrewegh A.A., Mahler M., Hedrich H.J., Haagmans B.L., Egberink H.F., Horzinek M.C., Rottier P.J., de Groot R.J. (1997). Persistence and evolution of feline coronavirus in a closed cat-breeding colony. Virology.

[30] Desmarets L.M., Vermeulen B.L., Theuns S., Conceicao-Neto N., Zeller M., Roukaerts I.D., Acar D.D., Olyslaegers D.A., Van Ranst M., Matthijnssens J. (2016). Experimental feline enteric coronavirus infection reveals an aberrant infection pattern and shedding of mutants with impaired infectivity in enterocyte cultures. Sci. Rep..

[31] Addie D.D., le Poder S., Burr P., Decaro N., Graham E., Hofmann-Lehmann R., Jarrett O., McDonald M., Meli M.L. (2015). Utility of feline coronavirus antibody tests. J. Feline Med. Surg..

[32] Lutz H., Gut M., Leutenegger C.M., Schiller I., Meli M.L. Inetics of FCoV Infection in Kittens Born in Catteries of High risk for FIP under Different Rearing Conditions. Proceedings of the Second International Feline Coronavirus/Feline Infectious Peritonitis Symposium.

[33] Hartmann K. (2005). Feline infectious peritonitis. Vet. Clin. N. Am. Small Anim. Pract..

[34] Rohner-Mächler M. (1999). Bestimmung der Ausscheidungskinetik von Felinen Coronaviren unter Feldbedingungen. Inaugural Thesis.

[35] Bell E.T., Toribio J.A., White J.D., Malik R., Norris J.M. (2006). Seroprevalence study of feline coronavirus in owned and feral cats in Sydney, Australia. Aust. Vet. J..

[36] Malbon A.J., Russo G., Burgener C., Barker E.N., Meli M.L., Tasker S., Kipar A. (2020). The Effect of Natural Feline Coronavirus Infection on the Host Immune Response: A Whole-Transcriptome Analysis of the Mesenteric Lymph Nodes in Cats with and without Feline Infectious Peritonitis. Pathogens.

[37] Mustaffa-Kamal F., Liu H., Pedersen N.C., Sparger E.E. (2019). Characterization of antiviral T cell responses during primary and secondary challenge of laboratory cats with feline infectious peritonitis virus (FIPV). BMC Vet. Res..

[38] Delaplace M., Huet H., Gambino A., Le Poder S. (2021). Feline Coronavirus Antivirals: A Review. Pathogens.

[39] Dickinson P.J., Bannasch M., Thomasy S.M., Murthy V.D., Vernau K.M., Liepnieks M., Montgomery E., Knickelbein K.E., Murphy B., Pedersen N.C. (2020). Antiviral treatment using the adenosine nucleoside analogue GS-441524 in cats with clinically diagnosed neurological feline infectious peritonitis. J. Vet. Intern. Med..

[40] Murphy B.G., Perron M., Murakami E., Bauer K., Park Y., Eckstrand C., Liepnieks M., Pedersen N.C. (2018). The nucleoside analog GS-441524 strongly inhibits feline infectious peritonitis (FIP) virus in tissue culture and experimental cat infection studies. Vet. Microbiol..

[41] Pedersen N.C., Kim Y., Liu H., Galasiti Kankanamalage A.C., Eckstrand C., Groutas W.C., Bannasch M., Meadows J.M., Chang K.O. (2018). Efficacy of a 3C-like protease inhibitor in treating various forms of acquired feline infectious peritonitis. J. Feline Med. Surg..

[42] Pedersen N.C., Perron M., Bannasch M., Montgomery E., Murakami E., Liepnieks M., Liu H. (2019). Efficacy and safety of the nucleoside analog GS-441524 for treatment of cats with naturally occurring feline infectious peritonitis. J. Feline Med. Surg..

[43] Krentz D., Zenger K., Alberer M., Felten S., Bergmann M., Dorsch R., Matiasek K., Kolberg L., Hofmann-Lehmann R., Meli M.L. (2021). Curing Cats with Feline Infectious Peritonitis with an Oral Multi-Component Drug Containing GS-441524. Viruses.

[44] Addie D.D., Covell-Ritchie J., Jarrett O., Fosbery M. (2020). Rapid Resolution of Non-Effusive Feline Infectious Peritonitis Uveitis with an Oral Adenosine Nucleoside Analogue and Feline Interferon Omega. Viruses.

[45] Addie D.D., Curran S., Bellini F., Crowe B., Sheehan E., Ukrainchuk L., Decaro N. (2020). Oral Mutian(R)X stopped faecal feline coronavirus shedding by naturally infected cats. Res. Vet. Sci..

[46] Chang H.W., Egberink H.F., Halpin R., Spiro D.J., Rottier P.J. (2012). Spike protein fusion peptide and feline coronavirus virulence. Emerg. Infect. Dis..

[47] Brunner C., Kanellos T., Meli M.L., Sutton D.J., Gisler R., Gomes-Keller M.A., Hofmann-Lehmann R., Lutz H. (2006). Antibody induction after combined application of an adjuvanted recombinant FeLV vaccine and a multivalent modified live virus vaccine with a chlamydial component. Vaccine.

[48] Lutz H., Hauser B., Horzinek M. (1984). On the serological diagnosis of feline infectious peritonitis. Prakt Tierarzt.

[49] Osterhaus A.D., Horzinek M.C., Reynolds D.J. (1977). Seroepidemiology of feline infectious peritonitis virus infections using transmissible gastroenteritis virus as antigen. Zent. Vet. B.

[50] Gut M., Leutenegger C.M., Huder J.B., Pedersen N.C., Lutz H. (1999). One-tube fluorogenic reverse transcription-polymerase chain reaction for the quantitation of feline coronaviruses. J. Virol. Methods.

[51] Tandon R., Cattori V., Gomes-Keller M.A., Meli M.L., Golder M.C., Lutz H., Hofmann-Lehmann R. (2005). Quantitation of feline leukaemia virus viral and proviral loads by TaqMan real-time polymerase chain reaction. J. Virol. Methods.

[52] Lutz M., Steiner A.R., Cattori V., Hofmann-Lehmann R., Lutz H., Kipar A., Meli M.L. (2020). FCoV Viral Sequences of Systemically Infected Healthy Cats Lack Gene Mutations Previously Linked to the Development of FIP. Pathogens.

[53] Kumar S., Stecher G., Li M., Knyaz C., Tamura K. (2018). MEGA X: Molecular Evolutionary Genetics Analysis across Computing Platforms. Mol. Biol. Evol..

[54] Thompson J.D., Higgins D.G., Gibson T.J. (1994). CLUSTAL W: Improving the sensitivity of progressive multiple sequence alignment through sequence weighting, position-specific gap penalties and weight matrix choice. Nucleic Acids Res..

[55] Rzhetsky A., Nei M. (1992). Statistical properties of the ordinary least-squares, generalized least-squares, and minimum-evolution methods of phylogenetic inference. J. Mol. Evol..

[56] Felsenstein J. (1985). Confidence Limits on Phylogenies: An Approach Using the Bootstrap. Evolution.

[57] Nei M., Kumar S. (2000). Molecular Evolution and Phylogenetics.

[58] Saitou N., Nei M. (1987). The neighbor-joining method: A new method for reconstructing phylogenetic trees. Mol. Biol. Evol..

[59] Chang H.W., de Groot R.J., Egberink H.F., Rottier P.J. (2010). Feline infectious peritonitis: Insights into feline coronavirus pathobiogenesis and epidemiology based on genetic analysis of the viral 3c gene. J. Gen. Virol..

[60] Barker E.N., Stranieri A., Helps C.R., Porter E.L., Davidson A.D., Day M.J., Knowles T., Kipar A., Tasker S. (2017). Limitations of using feline coronavirus spike protein gene mutations to diagnose feline infectious peritonitis. Vet. Res..

[61] Pedersen N.C., Liu H., Scarlett J., Leutenegger C.M., Golovko L., Kennedy H., Kamal F.M. (2012). Feline infectious peritonitis: Role of the feline coronavirus 3c gene in intestinal tropism and pathogenicity based upon isolates from resident and adopted shelter cats. Virus Res..

[62] Potten C.S. (1998). Stem cells in gastrointestinal epithelium: Numbers, characteristics and death. Philos. Trans. R. Soc. Lond. B Biol. Sci..

[63] Fish E.J., Diniz P.P.V., Juan Y.C., Bossong F., Collisson E.W., Drechsler Y., Kaltenboeck B. (2018). Cross-sectional quantitative RT-PCR study of feline coronavirus viremia and replication in peripheral blood of healthy shelter cats in Southern California. J. Feline Med. Surg..

[64] Doenges S.J., Weber K., Dorsch R., Fux R., Hartmann K. (2017). Comparison of real-time reverse transcriptase polymerase chain reaction of peripheral blood mononuclear cells, serum and cell-free body cavity effusion for the diagnosis of feline infectious peritonitis. J. Feline Med. Surg..

[65] Felten S., Leutenegger C.M., Balzer H.J., Pantchev N., Matiasek K., Wess G., Egberink H., Hartmann K. (2017). Sensitivity and specificity of a real-time reverse transcriptase polymerase chain reaction detecting feline coronavirus mutations in effusion and serum/plasma of cats to diagnose feline infectious peritonitis. BMC Vet. Res..

[66] Felten S., Weider K., Doenges S., Gruendl S., Matiasek K., Hermanns W., Mueller E., Matiasek L., Fischer A., Weber K. (2017). Detection of feline coronavirus spike gene mutations as a tool to diagnose feline infectious peritonitis. J. Feline Med. Surg..

[67] Pedersen N.C., Eckstrand C., Liu H., Leutenegger C., Murphy B. (2015). Levels of feline infectious peritonitis virus in blood, effusions, and various tissues and the role of lymphopenia in disease outcome following experimental infection. Vet. Microbiol..

[68] Longstaff L., Porter E., Crossley V.J., Hayhow S.E., Helps C.R., Tasker S. (2017). Feline coronavirus quantitative reverse transcriptase polymerase chain reaction on effusion samples in cats with and without feline infectious peritonitis. J. Feline Med. Surg..

[69] Stranieri A., Giordano A., Paltrinieri S., Giudice C., Cannito V., Lauzi S. (2018). Comparison of the performance of laboratory tests in the diagnosis of feline infectious peritonitis. J. Vet. Diagn. Investig..

[70] Jacobse-Geels H.E., Daha M.R., Horzinek M.C. (1982). Antibody, immune complexes, and complement activity fluctuations in kittens with experimentally induced feline infectious peritonitis. Am. J. Vet. Res..

[71] Olsen C.W., Corapi W.V., Ngichabe C.K., Baines J.D., Scott F.W. (1992). Monoclonal antibodies to the spike protein of feline infectious peritonitis virus mediate antibody-dependent enhancement of infection of feline macrophages. J. Virol..

[72] Pedersen N.C. (1987). Virologic and immunologic aspects of feline infectious peritonitis virus infection. Adv. Exp. Med. Biol..

[73] Belouzard S., Millet J.K., Licitra B.N., Whittaker G.R. (2012). Mechanisms of coronavirus cell entry mediated by the viral spike protein. Viruses.

[74] Millet J.K., Whittaker G.R. (2015). Host cell proteases: Critical determinants of coronavirus tropism and pathogenesis. Virus Res..

[75] Decaro N., Mari V., Lanave G., Lorusso E., Lucente M.S., Desario C., Colaianni M.L., Elia G., Ferringo F., Alfano F. (2021). Mutation analysis of the spike protein in Italian feline infectious peritonitis virus and feline enteric coronavirus sequences. Res. Vet. Sci..

[76] Porter E., Tasker S., Day M.J., Harley R., Kipar A., Siddell S.G., Helps C.R. (2014). Amino acid changes in the spike protein of feline coronavirus correlate with systemic spread of virus from the intestine and not with feline infectious peritonitis. Vet. Res..

[77] Jähne S., Felten S., Bergmann M., Erber K., Matiasek K., Meli M.L., Hofmann-Lehmann R., Hartmann K. (2022). Detection of feline coronavirus variants in cats without feline infectious peritonitis. Viruses.

[78] Brown M.A., Troyer J.L., Pecon-Slattery J., Roelke M.E., O’Brien S.J. (2009). Genetics and pathogenesis of feline infectious peritonitis virus. Emerg. Infect. Dis..

[79] Pedersen N.C., Liu H., Dodd K.A., Pesavento P.A. (2009). Significance of coronavirus mutants in feces and diseased tissues of cats suffering from feline infectious peritonitis. Viruses.

[80] Fischer Y., Ritz S., Weber K., Sauter-Louis C., Hartmann K. (2011). Randomized, placebo controlled study of the effect of propentofylline on survival time and quality of life of cats with feline infectious peritonitis. J. Vet. Intern. Med..

[81] Ritz S., Egberink H., Hartmann K. (2007). Effect of feline interferon-omega on the survival time and quality of life of cats with feline infectious peritonitis. J. Vet. Intern. Med..

