# Supplementary material for: Fecal Feline Coronavirus RNA Shedding and Spike Gene Mutations in Cats with Feline Infectious Peritonitis Treated with GS-441524
Source: Viruses. 2022 May 17;14(5):1069. doi: 10.3390/v14051069 (PMC9147249; doi:10.3390/v14051069)
Supplement: Supplementary file 1 [file viruses-14-01069-s001.zip › viruses-1680563-supplementary.pdf]

Supplementary

Table S1. Nucleotide sequences of the study in FASTA format.

>C2 Feces D143

TGGCATAATGGTTTTACCTGGTGTTGTGGATGCCAATAAAATGGCCATGTATACTGC
CTCATTAATAGGTGGCATGGCTATGGGTTCCATAACATCCTCTGTAGCTGTTCCTTTC
GCCATGCAAGTGCAGGCGAGGCTTAATTAA

>C4 Feces D135

TTGGCATAATGGTTTTACCTGGTGTTGTAGGACGAATAATAAGATGGCCATGTACAC
TGCCTCTCTWATAGGAGGTATGGCCATGGGCTCTATTACATCTGCTGTAGCAGGTCC
CTTTTGCTATGCAAGTGCAGGCGAGGCTTAATTAA

>C5 Feces D83

GCATAATGGTTTTACCTGGTGTTGTTGACCAAAACAAGATGGCTATGTACACTGCAT
CTTTAATAGGAGGTATGGCTATGGGCTCTATCACTTCTGCTGTAGCTGTCCCTTTTGC
CATGCAAGTGCAGGCGAGGCTTAATTA

>C16 Feces D56

CTTGGCATAATGGTTTTACCTGGTGTTGTGGATGACAATAAGATGGCCATGTACACTT
CCTCTTTAATAGGAGGTATGGCCATGGGCTCTATCACATCCGCTGTAGCTGTTCCTTT
CGCCATGCAAGTGCAGGCGAGGCTTAATT

>T2 Feces D0

TGGCATAATGGTTTTACCTGGTGTTGTGGATTCCAATAAAATGGCCATGTATACTGCC
TCATTAATAGGTGGCATGGCTATGGGTTCCATAACATCCTCTGTAGCTGTTCCTTTCG
CCATGCAAGTGCAGGCGAGGCTTAATTAA

>T2 Feces D1

GGCATAATGGTTTTACCTGGTGTTGTGGATTCCAATAAAATGGCCATGTATACTGCCT
CATTAATAGGTGGCATGGCTATGGGTTCCATAACATCCTCTGTAGCTGTTCCTTTCGC
CATGCAAGTGCAGGCGAGGCTTAATTAA

>T3 Effusion D1

GCATAATGGTTTTACCTGGTGTTGTAGACCAAAACAAAATGGCTATGTACACTGGCC
TCCTTTAATTGGCGGTATGGCTTTGGGCTCTATTACATCTGCTGTAGCTTGTTCCTTTT
GCCATGCAAGTGCAGGCGAGGCTTAATT

>T4 Effusion D0

GCATAATGGTTTTACCTGGTGTTGTAGACCAAAATAAGATGGCTATGTACACTGCCT
CTTTAATAGGAGGTATGGCCTTGGGTTCTATTACATNCTGCTGTAGCGGTCCTTTTTG
CTATGCAAGTGCAGGCGAGGCTTAATT

>T4 Feces D2

GCATAATGGTTTTACCTGGTGTTGTAGACCAAAATAAGATGGCTATGTACACTGCCT
CTTTAATAGGAGGTATGGCCATGGGTTCTATTACATCTGCTGTAGCGGTCCCTTTTGC
TATGCAAGTGCAGGCGAGGCTTAATT

>T5 Effusion D0

CAATGGCATAATGGTGCTACCGGGTGTTGTTGACCAAAACAAGATGGCTATGTACA
CTGCATCTTTAATAGGAGGTATGGCTYTGGGCTCTATCACTTCTGCTGTAGCTGTCCC
TTTTGCCATGCAAGTTCAGGCTAGGCTTAATTATGTTGCATTACAAACTGATGTGTTA
CAGGAAAACCAGAAGATACTTGCTAATGCTTTTAATAATGCTATTGGTAATATTACA
CTAGCGCTTGGAAAGGTTTCTAATGCTATTACAACCAYTTCAGCTGGTTTTAATACCA
TGGCCTCAGCACTGACCAAGATTCAGAGTGTAGTCAATCAACAAGGTGAGGCGTTG
AGTCAACTTACTAGTCAGYTACAGAAAAATTTCCAAGCTATTAGTAGTTCCATTGCT
GAAATCTACAATAGACTGGGAAAAGTGGAAGCTGATGCGCAAGTTGACCGCCTCAT
TACTGGCAGGTTGGCAGCACTTAATGCTTATGTGTCTCAAACTTTGACTCAGTATGCA
GAGGTCAAAGCTAGTAGACAACTGGCAATGGAGAAAGTTAATGAGTGTGTAAAATC
ACAGTCAGATAGGTATGGTTTCTGCGGGACTCGAGGGA

>T5 Effusion D1

GGCATAATGGTTTTACCTGGTGTTGTTGACCAAAACAAGATGGCTATGTACACTGCA
TCTTTAATAGGAGGTATGGCTCTGGGCTCTATCACTTCTGCTGTAGCTGTCCCTTTTGC
CATGCAAGTGCAGGCGAGGCTTAATT

>T5 Effusion D2

TCGGCATAATGGTTTTACCTGGTGTTGTTGACCAAAACAAGATGGCTATGTACACTG
CATCTTTAATAGGAGGTATGGCTCTGGGCTCTATCACTTCTGCTGTAGCTGTCCCTTTT
GCCATGCAAGTGCAGGCGAGGCTTAATT

>T5 Feces D1

TCGGGCATAATGGTTTTACCTGGTGTTGTTGACCAAAACAAGATGGCTATGTACACT
GCATCTTTAATAGGAGGTATGGCTATGGGCTCTATCACTTCTGCTGTAGCTGTCCCTT
TTGCCATGCAAGTGCAGGCGAGGCTTAATTA

>T5 Feces D2

ATGGTTTTACCTGGTGTTGTTGACCAAAACAAGAATGGCTATGTACACTGCATCTTTA
ATAGGAGGTATGGCTATGGGCTCTATCACTTCTGCTGTAGCTGTCCCTTTTGCCATGC
AAGTGCAGGCGAGGCTTAATTAT

>T7 Effusion D0

ACAATGGCATAATGGTTTTACCTGGTGTTGTAGATCAAAATAAGATGGCCATGTATA
CTGCCTCATTAATAGGCGGTATGGCATTGGGTTCTATTACATCTGCTGTAGCTGTTCC
TTTTGCTATGCAAGTGCAGGCTAGGCTTAATTATGTTGCATTGCAAACTGACGTTCTA
CAGGAAAACCAGAAAATACTTGCTAATGCCTTTAATAATGCCATTGGTAACATTACA
CTAGCGCTTGGAAAAGTTTCTAACGCTATTACAACTACTTCAGAGGGATTTAACAGT
ATGGCCTCAGCACTGACTAAGATTCAAAGTGTAGTTAATCAACAAGGTGAAGCGTT
GAGTCAACTTACTAGCCAGTTACAGAAAAACTTCCAGGCTATTAGTAGTTCTATTGC
TGAAATCTACAATAGACTGGAAAAAGCGGAAGCAGATGCACAAGTTGACCGTCTCA
TTACTGGTAGATTGGCAGCACTTAATGCCTATGTGTCTCAAACTTTAACTCAGTATGC
TGAAGTCAAGGCTAGCAGACAACTTGCAATGGAGAAAGTTAATGAGTGTGTGAAAT
CACAGTCTGATAGGTATGGTTTCTGCGGGACCTCGAGGGA

>T7 Feces D2

GGCATAATGGTTTTACCTGGTGTTGTAGATCAAAATAAGATGGCCAATGTATACTGC
CTCATTAATAGGCGGTATGGCTATGGGTTCTATTACATCTGCTGTAGCTGTTCCTTTTG
CTATGCAAGTGCAGGCGAGGCTTAATTAA

>T7 Feces D3

AATGGTTTTACCTGGTGTTGTAGATCAAAATAAGATGGCCATGTATACTGCCTCATTT
AATAGGCGGTATGGCTATGGGTTCTATTACATCTGCTGTAGCTGTTCCTTTTGCTATG
CAAGTGCAGGCGAGGCTTAATT

>T8 Effusion D0

CAATGGCATAATGGTTTTACCTGGCGTCGTAGATTATAATAAGATGGCCATGTACAC
AGCCTCTTTAATAGGAGGTATGGCTTTGGGTTCTATTACATCTGCTGTAGCCGTTCCC
TTTGCTATGCAAGTGCAGGCTAGGCTTAATTATGTTGCATTGCAAACGGATGTATTAC
AGGAAAACCAGAAAATACTTGCTAATGCCTTTAATAATGCCATTGGTAATATTACAC
TAGCACTTGGAAAGGTTTCTAATGCCATTACAACTATTTCAGATGGATTTAATACTAT
GGCTTCAGCATTAACTAAGATCCAGAGTGTGGTTAATCAACAGGGTGAAGCGTTGA
GTCAACTTACCAGTCAGTTACAGAAAAACTTCCAGGCTATTAGTAGTTCCATTGCTG
AAATCTACAATAGATTGGAAAAGGTGGAAGCTGATGCGCAAGTTGACCGTCTCATT
ACTGGAAGATTGGCAGCACTTAATGCTTATGTATCTCAAACTTTAACTCAGTATGCT
GAAGTCAAGGCTAGTAGGCAACTGGCAATGGAAAAAGTTAATGAGTGTGTCAAATC
GCAGTCGGATAGGTATGGTTTCTGCGGGACTCGAGGGA

>T11 Effusion D0

GGCATAATGGTTTTACCTGGCGTTGCAGATGCTAATAAGATGGCCATGTATACTGCT
TCTCTAATAGGAGGTATGGCCTTTGGCTCTATTACATCTGCTGCGGCTGTTCCCTTTGC
CATGCAAGTGCAGGCGAGGCTTAATTAA

>T11 Effusion D0 c1

GGCATAATGGTTTTACCTGGCGTTGCAGATGCTAATAAGATGGCCATGTATACTGCT
TCTCTAATAGGAGGTATGGCCTTTGGCTCTATTACATCTGCTGCGGCTGTTCCCTTTGC
CATGCAAGTGCAGGCGAGGCTTAATT

>T11 Effusion D0 c2

GGCATAATGGTTTTACCTGGCGTTGCAGATGCTAATAAGATGGCCATGTATACTGCT
TCTCTAATAGGAGGTATGGCCTTTGGCTCTATTACATCTGCTGCGGCTGTTCCCTTTGC
CATGCAAGTGCAGGCGAGGCTTAATTA

>T11 Effusion D0 c3

GGCATAATGGTTTTACCTGGCGTTGCAGATGCTAATAAGATGGCCATGTATACTGCT
TCTCTAATAGGAGGTATGGCCTTTGGCTCTATTACATCTGCTGCGGCTGTTCCCTTTGC
CATGCAAGTGCAGGCGAGGCTTAATTA

>T11 Effusion D0 c4

GGCATAATGGTTTTACCTGGCGTTGCAGATGCTAATAAGATGGCCATGTATACTGCT
TCTCTAATAGGAGGTATGGCCTTTGGCTCTATTACATCTGCTGCGGCTGTTCCCTTTGC
CATGCAAGTGCAGGCGAGGCTTAATA

>T11 Effusion D0 c6

GGCATAATGGTTTTACCTGGCGTTGCAGATGCTAATAAGATGGCCATGTATACTGCT
TCTCTAATAGGAGGTATGGCCTTTGGCTCTATTACATCTGCTGCGGCTGTTCCCTTTGC
CATGCAAGTGCAGGCGAGGCTTAATTA

>T11 Effusion D0 c7

GGCATAATGGTTTTACCTGGCGTTGCAGATGCTAATAAGATGGCCATGTATACTGCT
TCTCTAATAGGAGGTATGGCCTTTGGCTCTATTACATCTGCTGCGGCTGTTCCCTTTGC
CATGCAAGTGCAGGCGAGGCTTAATT

>T11 Effusion D0 c8

GGCATAATGGTTTTACCTGGCGTTGCAGATGCTAATAAGATGGCCATGTATACTGCT
TCTCTAATAGGAGGTATGGCCTTTGGCTCTATTACATCTGCTGCGGCTGTTCCCTTTGC
CATGCAAGTGCAGGCGAGGCTTAATTA

>T11 Effusion D0 c9

GGCATAATGGTTTTACCTGGCGTTGCAGATGCTAATAAGATGGCCATGTATACTGCT
TCTCTAATAGGAGGTATGGCCTTTGGCTCTATTACATCTGCTGCGGCTGTTCCCTTTGC
CATGCAAGTGCAGGCGAGGCTTAATTA

>T11 Effusion D0 c10

GGCATAATGGTTTTACCTGGCGTTGCAGATGCTAATAAGATGGCCATGTATACTGCT
TCTCTAATAGGAGGTATGGCCTTTGGCTCTATTACATCTGCTGCGGCTGTTCCCTTTGC
CATGCAAGTGCAGGCGAGGCTTAATT

>T11 Effusion D1

ATAATGGTTTTACCTGGCGTTGCAGATGCTAATAAGATGGCCATGTATACTGCTTCTC
TAATAGGAGGTATGGCCTTTGGCTCTATTACATCTGCTGCGGCTGTTCCCTTTGCCAT
GCAAGTGCAGGCGAGGCTTAATTAA

>T11 Effusion D1 c1

GGCATAATGGTTTTACCTGGCGTTGCAGATGCTAATAAGATGGCCATGTATACTGCT
TCTCTAATAGGAGGTATGGCCTTTGGCTCTATTACATCTGCTGCGGCTGTTCCCTTTGC
CATGCAAGTGCAGGCGAGGCTTAATTA

>T11 Effusion D1 c2

GGCATAATGGTTTTACCTGGCGTTGCAGATGCTAATAAGATGGCCATGTATACTGCT
TCTCTAATAGGAGGTATGGCCTTTGGCTCTATTACATCTGCTGCGGCTGTTCCCTTTGC
CGTGCAAGTGCAGGCGAGGCTTAATTA

>T11 Effusion D1 c3

GGCATAATGGTTTTACCTGGCGTTGCAGATGCTAATAAGATGGCCATGTATACTGCT
TCTCTAATAGGAGGTATGGCCTTTGGCTCTATTACATCTGCTGCGGCTGTTCCCTTTGC
CATGCAAGTGCAGGCGAGGCTTAATTA

>T11 Effusion D1 c4

GGCATAATGGTTTTACCTGGCGTTGCAGATGCTAATAAGATGGCCATGTATACTGCT
TCTCTAATAGGAGGTATGGCCTTTGGCTCTATTACATCTGCTGCGGCTGTTCCCTTTGC
CATGCAAGTGCAGGCGAGGCTTAATTA

>T11 Effusion D1 c5

GGCATAATGGTTTTACCTGGCGTTGCAGATGCTAATAAGATGGCCATGTATACTGCT
TCTCTAATAGGAGGTATGGCCTTTGGCTCTATTACATCTGCTGCGGCTGTTCCCTTTGC
CATGCAAGTGCAGGCGAGGCTTAATTA

>T11 Effusion D1 c6

GGCATAATGGTTTTACCTGGCGTTGCAGATGCTAATAAGATGGCCATGTATACTGCT
TCTCTAATAGGAGGTATGGCCTTTGGCTCTATTACATCTGCTGCGGCTGTTCCCTTTGC
CATGCAAGTGCAGGCGAGGCTTAATTA

>T11 Effusion D1 c7

GGCATAATGGTTTTACCTGGCGTTGCAGATGCTAATAAGATGGCCATGTATACTGCT
TCTCTAATAGGAGGTATGGCCTTTGGCTCTATTACATCTGCTGCGGCTGTTCCCTTTGC
CATGCAAGTGCAGGCGAGGCTTAATTA

>T11 Effusion D1 c8

GGCATAATGGTTTTACCTGGCGTTGCAGATGCTAATAAGATGGCCATGTATACTGCT
TCTCTAATAGGAGGTATGGCCTTTGGCTCTATTACATCTGCTGCGGCTGTTCCCTTTGC
CATGCAAGTGCAGGCGAGGCTTAATTA

>T11 Effusion D1 c9

GGCATAATGGTTTTACCTGGCGTTGCAGATGCTAATAAGATGGCCATGTATACTGCT
TCTCTAATAGGAGGTATGGCCTTTGGCTCTATTACATCTGCTGCGGCTGTTCCCTTTGC
CATGCAAGTGCAGGCGAGGCTTAATTA

>T11 Effusion D1 c10

GGCATAATGGTTTTACCTGGCGTTGCAGATGCTAATAAGATGGCCATGTATACTGCT
TCTCTAATAGGAGGTATGGCCTTTGGCTCTATTACATCTGCTGCGGCTGTTCCCTTTGC
CATGCAAGTGCAGGCGAGGCTTAATTA

>T12 Effusion D0

GGCATAATGGTTTTACCTGGTGTTGTTGATCAAAATAAAATGGCTATGTACACTTCCT
CTTTAATAGGAGGTATGGCCATGGGTGCCATCACGTCCGCTGTAGCAGTCCCTTTCG
CCATGCAAGTGCAGGCGAGGCTTAAT

>T12 Effusion D0 c1

GGCATAATGGTTTTACCTGGTGTTGTTGATCAAAATAAAATGGCTATGTACACTTCCT
CTTTAATAGGAGGTATGGCCATGGGTGCCATCACGTCCGCTGTAGCAGTCCCTTTCG
CCATGCAAGTGCAGGCGAGGCTTAATTA

>T12 Effusion D0 c3

GGCATAATGGTTTTACCTGGTGTTGTTGATCAAAATAAAATGGCTATGTACACTTCCT
CTTTAATAGGAGGTATGGCCATGGGTGCCATCACGTCCGCTGTAGCAGTCCCTTTCG
CCATGCAAGTGCAGGCGAGGCTTAATTA

>T12 Effusion D0 c4

GGCATAATGGTTTTACCTGGTGTTGTTGATCAAAATAAAATGGCTATGTACACTTCCT
CTTTAATAGGAGGTATGGCCATGGGTGCCATCACGTCCGCTGTAGCAGTCCCTTTCG
CCATGCAAGTGCAGGCGAGGCTTAATTA

>T12 Effusion D0 c5

GGCATAATGGTTTTACCTGGTGTTGTTGATCAAAGTAAAATGGCTATGTACACTTCCT
CTTTAATAGGAGGTATGGCCATGGGTGCCATCACGTCCGCTGTAGCAGTCCCTTTCG
CCATGCAAGTGCAGGCGAGGCTTAATTA

>T12 Effusion D0 c6

GGCATAATGGTTTTACCTGGTGTTGTTGATCAAAATAAAATGGCTATGTACACTTCCT
CTTTAATAGGAGGTATGGCCATGGGTGCCATCACGTCCGCTGTAGCAGTCCCTTTCG
CCATGCAAGTGCAGGCGAGGCTTAATTA

>T12 Effusion D0 c7

GGCATAATGGTTTAACCTGGTGTTGTTGATCAAAATAAAATGGCTATGTACACTTCCT
CTTTAATAGGAGGTATGGCCATGGGTGCCATCACGTCCGCTGTAGCAGTCCCTTTCG
CCATGCAAGTGCAGGCGAGGCTTAATTA

>T12 Effusion D0 c8

GGCATAATGGTTTTACCTGGTGTTGTTGATCAAAATAAAATGGCTATGTACACTTCCT
CTTTAATAGGAGGTATGGCCATGGGTGCCATCACGTCCGCTGTAGCAGTCCCTTTCG
CCATGCAAGTGCAGGCGAGGCTTAATTA

>T12 Effusion D0 c9

GGCATAATGGTTTTACCTGGTGTTGTTGATCAAGATAAAATGGCTATGTACACTTCCT
CTTTAATAGGAGGTATGGCCATGGGTGCCATCACGTCCGCTGTAGCAGTCCCTTTCG
CCATGCAAGTGCAGGCGAGGCTTAA

>T12 Effusion D0 c10

GGCATAATGGTTTTACCTGGTGTTGTTGATCAAAATAAAATGGCTATGTACACTTCCT
CTTTAATAGGAGGTATGGCCATGGGTGCCATCACGTCCGCTGTAGCAGTCCCTTTCG
CCATGCAAGTGCAGGCGAGGCTTAATTA

>T14 Blood D0

GCATAATGGTTTTACCGGCGTTGTGGATGCCAATAAGATGGCCATGTACACCGCCTC
TTTGATAGGAGGTATGGCTATGGGTGCCATTACCTCCGCTGTAGCTGTTCCTTTTGCT
ATGCAAGTGCAGGCGAGGCTTAATTAAA

>T14 Blood D0 c2

GGCATAATGGTTTTACCTGGCGTTGTGGATGCCAATAAGATGGCCATGTACACCGCC
TCTTTGATAGGAGGTATGGCTATGGGTGCCATTACCTCCGCTGTAGCTGTTCCTTTTG
CTATGCAAGTGCAGGCGAGGCTTAATTA

>T14 Blood D0 c3

GGCATAATGGTTTTACCTGGCGTTGTGGATGCCAATAAGATGGCCATGTACACCGCC
TCTTTGATAGGAGGTATGGCTATGGGTGCCATTACCTCCGCTGTAGCTGTTCCTTTTG
CTATGCAAGTGCAGGCGAGGCTTAATTA

>T14 Blood D0 c5

GGCATAATGGTTTTACCTGGCGTTGTGGATGCCAATAAGATGGCCATGTACACCGCC
TCTTTGATAGGAGGTATGGCTATGGGTGCCATTACCTCCGCTGTAGCTGTTCCTTTTG
CTATGCAAGTGCAGGCGAGGCTTAATTT

>T14 Blood D0 c6

GGCATAATGGTTTTACCTGGCGTTGTGGATGCCAATAAGATGGCCATGTACACCGCC
TCTTTGATAGGAGGTATGGCTATGGGTGCCATTACCTCCGCTGTAGCTGYTCCTTTTG
CTATGCAAGTGCAGGCGAGGCTTAATTA

>T14 Blood D0 c8

GGCATAATGGTTTTACCTGGCGTTGTGGATGCCAATAAGATGGCCATGTACACCGCC
TCTTTGATAGGAGGTATGGCTATGGGTGCCATTACCTCCGCTGTAGCTGTTCCTTTTG
CTATGCAAGTGCAGGCGAGGCTTAATTT

>T14 Blood D0 c10

GGCATAATGGTTTTACCTGGCGTTGTGGATGCCAATAAGATGGCCATGTACACCGCC
TCTTTGATAGGAGGTATGGCTATGGGTGCCATTACCTCCGCTGTAGCTGTTCCTTTTG
CTATGCAAGTGCAGGCGAGGCTTAATTA

>T14 Effusion D0

CATAATGGTTTTACCGGCGTTGTGGATGCCAATAAGATGGCCATGTACACCGCCTCT
TTGATAGGAGGTATGGCTATGGGTGCCATTACCTCCGCTGTAGCTGTTCCTTTTGCTA
TGCAAGTGCAGGCGAGGCTTAATTAA

>T14 Effusion D0 c1

GGCATAATGGTTTTACCTGGCGTTGTGGATGCCAATAAGATGGCCATGTACACCGCC
TCTTTGATAGGAGGTATGGCTATGGGTGCCATTACCTCCGCTGTAGCTGTTCCTTTTG
CTATGCAAGTGCAGGCGAGGCTTAATTA

>T14 Effusion D0 c2

GGCATAATGGTTTTACCTGGCGTTGTGGATGCCAATAAGATGGCCATGTACACCGCC
TCTTTGATAGGAGGTATGGCTATGGGTGCCATTACCTCCGCTGTAGCTGTTCCTTTTG
CTATGCAAGTGCAGGCGAGGCTTAATTA

>T14 Effusion D0 c3

GGCATAATGGTTTTACCTGGCGTTGTGGATGCCAATAAGATGGCCATGTACACCGCC
TCTTTGATAGGAGGTATGGCTATGGGTGCCATTACCTCCGCTGTAGCTGTTCCTTTTG
CTATGCAAGTGCAGGCGAGGCTTAATT

>T14 Effusion D0 c4

GGCATAATGGTTTTACCTGGCGTTGTGGATGCCAATAAGATGGCCATGTACACCGCC
TCTTTGATAGGAGGTATGGCTATGGGTGCCATTACCTCCGCTGTAGCTGTTCCTTTTG
CTATGCAAGTGCAGGCGAGGCTTAATTA

>T14 Effusion D0 c5

GGCATAATGGTTTTACCTGGCGTTGTGGATGCCAATAAGATGGCCATGTACACCGCC
TCTTTGATAGGAGGTATGGCTATGGGTGCCATTACCTCCGCTGTAGCTGTTCCTTTTG
CTATGCAAGTGCAGGCGAGGCTTAATT

>T14 Effusion D0 c7

GGCATAATGGTTTTACCTGGCGTTGTGGATGCCAATAAGATGGCCATGTACACCGCC
TCTTTGATAGGAGGTATGGCTATGGGTGCCATTACCTCCGCTGTAGCTGTTCCTTTTG
CTATGCAAGTGCAGGCGAGGCTTAATT

>T14 Effusion D0 c8

GGCATAATGGTTTTACCTGGCGTTGTGGATGCCAATAAGATGGCCATGTACACCGCC
TCTTTGATAGGAGGTATGGCTATGGGTGCCATTACCTCCGCTGTAGCTGTTCCTTTTG
CTATGCAAGTGCAGGCGAGGCTTAATTA

>T14 Effusion D0 c9

GGCATAATGGTTTTACCTGGCGTTGTGGATGCCAATAAGATGGCCATGTACACCGCC
TCTTTGATAGGAGGTATGGCTATGGGTGCCATTACCTCCGCTGTAGCTGTTCCTTTTG
CTATGCAAGTGCAGGCGAGGCTTAATTA

>T14 Effusion D0 c10

GGCATAATGGTTTTACCTGGCGTTGTGGATGCCAATAAGATGGCCATGTACACCGCC
TCTTTGATAGGAGGTATGGCTATGGGTGCCATTACCTCCGCTGTAGCTGTTCCTTTTG
CTATGCAAGTGCAGGCGAGGCTTAATTA

>T14 Effusion D1

GGCATAATGGTTTTACCGGCGTTGTGGATGCCAATAAGATGGCCATGTACACCGCCT
CTTTGATAGGAGGTATGGCTATGGGTGCCATTACCTCCGCTGTAGCTGTTCCTTTTGC
TATGCAAGTGCAGGCGAGGCTTAATTAA

>T14 Effusion D1 c1

GGCATAATGGTTTTACCTGGCGTTGTGGATGCCAATAAGATGGCCATGTACACCGCC
TCTTTGATAGGAGGTATGGCTATGGGTGCCATTACCTCCGCTGTAGCTGTTCCTTTTG
CTATGCAAGTGCAGGCGAGGCTTAATTA

>T14 Effusion D1 c2

GGCATAATGGTTTTACCTGGCGTTGTGGATGCCAATAAGATGGCCATGTACACCGCC
TCTTTGATAGGAGGTATGGCTATGGGTGCCATTACCTCCGCTGTAGCTGTTCCTTTTG
CTATGCAAGTGCAGGCGAGGCTTAATTA

>T14 Effusion D1 c3

GGCATAATGGTTTTACCTGGCGTTGTGGATGCCAATAAGATGGCCATGTACACCGCC
TCTTTGATAGGAGGTATGGCTATGGGTGCCATTACCTCCGCTGTAGCTGTTCCTTTTG
CTATGCAAGTGCAGGCGAGGCTTAATTA

>T14 Effusion D1 c4

GGCATAATGGTTTTACCTGGCGTTGTGGATGCCAATAAGATGGCCATGTACACCGCC
TCTTTGATAGGAGGTATGGCTATGGGTGCCATTACCTCCGCTGTAGCTGTTCCTTTTG
CTATGCAAGTGCAGGCGAGGCTTAATTA

>T14 Effusion D1 c5

GGCATAATGGTTTTACCTGGCGTTGTGGATGCCAATAAGATGGCCATGTACACCGCC
TCTTTGATAGGAGGTATGGCTATGGGTGCCATTACCTCCGCTGTAGCTGTTCCTTTTG
CTATGCAAGTGCAGGCGAGGCTTAATTA

>T14 Effusion D1 c6

GGCATAATGGTTTTACCTGGCGTTGTGGATGCCAATAAGATGGCCATGTACACCGCC
TCTTTGATAGGAGGTATGGCTATGGGTGCCATTACCTCCGCTGTAGCTGTTCCTTTTG
CTATGCAAGTGCAGGCGAGGCTTAATTA

>T14 Effusion D1 c8

GGCATAATGGTTTTACCTGGCGTTGTGGATGCCAATAAGATGGCCATGTACACCGCC
TCTTTGATAGGAGGTATGGCTATGGGTGCCATTACCTCCGCTGTAGCTGTTCCTTTTG
CTATGCAAGTGCAGGCGAGGCTTAATTA

>T14 Effusion D1 c9

GGCATAATGGTTTTACCTGGCGTTGTGGATGCCAATAAGATGGCCATGTACACCGCC
TCTTTGATAGGAGGTATGGCTATGGGTGCCATTACCTCCGCTGTAGCTGTTCCTTTTG
CTATGCAAGTGCAGGCGAGGCTTAATTA

>T14 Effusion D1 c10

GGCATAATGGTTTTACCTGGCGTTGTGGATGCCAATAAGATGGCCATGTACACCGCC
TCTTTGATAGGAGGTATGGCTATGGGTGCCATTACCTCCGCTGTAGCTGTTCCTTTTG
CTATGCAAGTGCAGGCGAGGCTTAATTA

>T15 Effusion D0

TCGGGCATAATGGTTTTACCTGGCGTCGTAGATGGTAACAAGATGGCCATGTACACA
GCCTCTTTAATAGGAGGTATGGCTTTGGGTTCTATTACATCTGCTGTAGCCGTTCCCTT
TGCTATGCAAGTGCAGGCGAGGCTTAATTAAA

>T15 Feces D0

TCGGCATAATGGTTTTACCTGGCGTCGTAGATGATAACAAGATGGCCATGTACACAG
CCTCTTTAATAGGAGGTATGGCTATGGGTTCTATTACATCTGCTGTAGCCGTTCCCTTT
GCTATGCAAGTGCAGGCGAGGCTTAATTA

>T16 Feces D0

GCATAATGGTTTTACCTGGTGTTGCAGATCAAAATAAGATGGCTATGTACACTGCCT
CTTTAATAGGAGGTATGGCCATGGGCTCTATCACATTCCGCTGTAGCTGTTCCTTTCG
CCATGCAAGTGCAGGCGAGGCTTAATTAAA

>T16 Feces D1

GCATAATGGTTTTACCTGGTGTTGCAGATCAAAATAAGATGGCTATGTACACTGCCT
CTTTAATAGGAGGTATGGCCATGGGCTCTATCACATCCGCTGTAGCTGTTCCTTTCGC
CATGCAAGTGCAGGCGAGGCTWAATTAAAARR

>T18 Effusion D0

GGCATAATGGTTTTACCTGGTGTTGTAGATTATAACAAAATGGCTATGTACACTGCCT
CTTTAATAGGCGGTATGGCTTTGGGTTCCTATTACGTCTGCAGTAGCTGTCCCTTTTGC
CATGCAAGTGCAGGCGAGGCTTAATT

>T18 Effusion D1

GCATAATGGTTTTACCTGGTGTTGTAGATTATAACAAAATGGCTATGTACACTGCCTC
TTTAATAGGCGGTATGGCTTTGGGTTCTATTACGTCTGCAGTAGCTGTCCCTTTTGCCATGCAA
GTGCAGGCGAGGCTTAATT
